# A new gene expression signature, the ClinicoMolecular Triad Classification, may improve prediction and prognostication of breast cancer at the time of diagnosis

**DOI:** 10.1186/bcr3017

**Published:** 2011-09-22

**Authors:** Dong-Yu Wang, Susan J Done, David R McCready, Scott Boerner, Supriya Kulkarni, Wey Liang Leong

**Affiliations:** 1 Ontario Cancer Institute and Campbell Family Institute of Cancer Research, Princess Margaret Hospital, University Health Network, University of Toronto, 610 University Avenue, Toronto, ON, M5G 2M9, Canada; 2 Department of Pathology, Princess Margaret Hospital, University Health Network, University of Toronto, 610 University Avenue, Toronto, ON, M5G 2M9, Canada; 3 Department of General Surgery and Surgical Oncology, Princess Margaret Hospital, University Health Network, University of Toronto, 610 University Avenue, Toronto, ON, M5G 2M9, Canada; 4 Department of Radiology, Princess Margaret Hospital, University Health Network, University of Toronto, 610 University Avenue, Toronto, ON, M5G 2M9, Canada

## Abstract

**Introduction:**

When making treatment decisions, oncologists often stratify breast cancer (BC) into a low-risk group (low-grade estrogen receptor-positive (ER+)), an intermediate-risk group (high-grade ER+) and a high-risk group that includes Her2+ and triple-negative (TN) tumors (ER-/PR-/Her2-). None of the currently available gene signatures correlates to this clinical classification. In this study, we aimed to develop a test that is practical for oncologists and offers both molecular characterization of BC and improved prediction of prognosis and treatment response.

**Methods:**

We investigated the molecular basis of such clinical practice by grouping Her2+ and TN BC together during clustering analyses of the genome-wide gene expression profiles of our training cohort, mostly derived from fine-needle aspiration biopsies (FNABs) of 149 consecutive evaluable BC. The analyses consistently divided these tumors into a three-cluster pattern, similarly to clinical risk stratification groups, that was reproducible in published microarray databases (*n *= 2,487) annotated with clinical outcomes. The clinicopathological parameters of each of these three molecular groups were also similar to clinical classification.

**Results:**

The low-risk group had good outcomes and benefited from endocrine therapy. Both the intermediate- and high-risk groups had poor outcomes, and their BC was resistant to endocrine therapy. The latter group demonstrated the highest rate of complete pathological response to neoadjuvant chemotherapy; the highest activities in Myc, E2F1, Ras, β-catenin and IFN-γ pathways; and poor prognosis predicted by 14 independent prognostic signatures. On the basis of multivariate analysis, we found that this new gene signature, termed the "ClinicoMolecular Triad Classification" (CMTC), predicted recurrence and treatment response better than all pathological parameters and other prognostic signatures.

**Conclusions:**

CMTC correlates well with current clinical classifications of BC and has the potential to be easily integrated into routine clinical practice. Using FNABs, CMTC can be determined at the time of diagnostic needle biopsies for tumors of all sizes. On the basis of using public databases as the validation cohort in our analyses, CMTC appeared to enable accurate treatment guidance, could be made available in preoperative settings and was applicable to all BC types independently of tumor size and receptor and nodal status. The unique oncogenic signaling pathway pattern of each CMTC group may provide guidance in the development of new treatment strategies. Further validation of CMTC requires prospective, randomized, controlled trials.

## Introduction

The presence of estrogen receptor (ER), progesterone receptor (PR) and human epidermal growth factor receptor 2 (Her2, also known as *ERBB2*) is routinely reported in the pathological assessment of breast cancer. These three receptors have become the mainstay of clinical and molecular classification of breast cancer [1,2]. In general, positive ER and PR status (ER+ and PR+, respectively) are considered good prognostic indicators, whereas positive Her2 status is considered a poor prognostic indicator [2]. However, negative status in all three receptors, that is, ER-, PR- and Her2-, also referred as "triple-negative" (TN) status, is also considered a poor prognostic indicator [3]. Because most basal-like subtype tumors are TN, these terms have been used interchangeably, but in actual fact TN and basal-like breast cancer are not the same and some of them can be differentiated from each other by more in-depth molecular characterization [3-5]. Oncologists generally divide breast cancer into three clinically relevant groups when making treatment decisions. Group 1 breast cancers are generally low-risk and ER+ and respond well to endocrine therapy (ET), such as tamoxifen. Group 2 breast cancers are ER+ but carry a poor prognosis despite ET, and therefore chemotherapy is strongly recommended for patients in this group. Group 3 breast cancers are ER-, including Her2+ and TN cancers with a poor prognosis that generally improves with chemotherapy, as well as trastuzumab if necessary.

There is some indirect evidence that supports stratifying Her2+ and TN breast cancer into the same high-risk group. There is no significant difference in the clinical outcomes of patients with the basal-like and Her2+ subtypes of breast cancer [5-7]. Even though there is no standard targeted systemic therapy for TN tumors [3,4,8], such as trastuzumab for Her2+ tumors [9], the rates of complete clinical response and complete pathological response (pCR) to neoadjuvant chemotherapies are also similar in both Her2+ and TN breast cancer [10-12]. Recently, investigators in both the CALGB 9840 trial [13] and the NSABP-B31 trial [14,15] reported responses of some Her2- breast cancers to trastuzumab and raised some controversies about the classification of breast cancer. Indirectly, these studies suggest that Her2+ breast cancer may not be as different from TN breast cancer as previously thought. Moreover, a relatively high proportion of TN tumors have genomic profiles similar to those of Her2+ tumors [16].

In the early 2000s, Perou and colleagues [6,7,17] reported the intrinsic gene expression profile that divides breast tumors into five or more molecular subtypes. More recently, on the basis of oncogenic pathway activity analysis, a more extensive classification with up to 18 subtypes for breast cancer was reported [18]. It remains a major challenge to use these molecular profiles to guide clinical treatment decisions [19] as they become increasingly complex for patients and clinicians alike and do not correlate with how breast cancer is clinically classified. On the other hand, many prognostic gene expression signatures that dichotomize selected patient populations into good and poor prognosis groups [20] lack the specificity to provide guidance on various treatment options.

In this study, we aimed to develop a molecular test that can be used preoperatively to guide treatment decisions, such as whether to initiate neoadjuvant therapy. For that reason, we decided to collect most of our clinical specimens by fine-needle aspiration biopsy (FNAB) taken from consecutive suspicious breast tumors at the time of clinical diagnostic core biopsy. Our study included relatively small breast cancers that had been routinely excluded in previous studies in which fresh surgical specimens or banked tissues were examined. After confirming the clinical diagnoses and the presence of tumor cells in the samples, gene profiles were generated from FNAB specimens by using a commercially available genome-wide microarray platform. To keep the molecular profiles clinically relevant, we asked whether there is a molecular basis for the clinical practice of lumping Her2+ and TN breast cancers together into the same high-risk group. We analysed the molecular phenotype of Her2+/TN breast cancers and developed a novel gene signature, termed the "ClinicoMolecular Triad Classification" (CMTC), which divides all breast cancers into three groups similar to the three risk groups that oncologists refer to. Each CMTC group displayed a unique pattern of oncogenic signaling pathway activities. To determine the clinical significance of the CMTC classification scheme, we correlated the three CMTC groups using standard pathology parameters, and the results were reproduced in a large independent validation cohort. Using multivariate analyses, CMTC was the best among 14 published prognostic gene signatures and clinical receptor statuses in predicting breast cancer recurrence and treatment response.

## Materials and methods

### Patients and samples

The primary data set consisted of 161 prospectively recruited, consecutive surgical patients with breast tumors. A total of 172 tissue samples were collected at the University Health Network (UHN) and Mount Sinai Hospital (MSH), Toronto, ON, Canada. We excluded samples from five benign tumors, five ductal carcinoma *in situ *samples and two with a low RNA integrity number (RIN). That left 149 invasive breast cancers used as the training cohort, including 121 FNABs, 10 core biopsies and 18 fresh frozen tissue specimens from the BioBank at UHN (Toronto, ON, Canada). FNABs were obtained by passing a 25-gauge needle into the tumor 10 to 20 times with suction using a 10-ml syringe. The cells were suspended in CytoLyt solution (Cytyc Corp, Marlborough, MA, USA) with an aliquot (10% vol/vol) sent for cytological analysis by a cytopathologist (SB). All FNAB samples had 80% or more malignant cells to be included in this study. The remaining cells were centrifuged and resuspended in 500 μl of RNA extraction lysis buffer (Qiagen, Valencia, CA, USA), then snap-frozen to -80°C for later processing. Core biopsies were taken by our radiologist (SK) at the time of diagnostic procedures. This study was approved by the Research Ethics Boards at our institutions (UHN and MSH). All patients were recruited prospectively and gave their written informed consent to participate in the study. The clinical follow-up data were collected until April 2010 with median follow-up of 31 months. The information for the 149 patients is provided in Table S1 in Additional file 1.

### RNA extraction and microarray process

After we determined that the tissue samples satisfied cytological criteria, the frozen FNAB lysates were thawed and RNA was extracted using the RNeasy Micro and RNeasy Mini kits (Qiagen) for FNABs and core biopsies and UHN BioBank samples, respectively, according to the manufacturer's protocols. The quality and quantity of the RNA were analyzed using an Agilent 2100 Bioanalyzer (Agilent Technologies, Palo Alto, CA, USA), and only the samples with a RIN higher than 5.5 were used in this study. The DNA microarray analyses were then performed according to the Illumina Whole-Genome Gene Expression direct hybridization assay protocols (Illumina Inc, San Diego, CA, USA) at The Centre of Applied Genomics (Toronto, ON, Canada). Briefly, 250 ng of total RNA were reverse-transcribed into cDNA, followed by *in vitro *transcription amplification to generate biotin-labeled cRNA using the Ambion TotalPrep RNA Amplification Kit (Applied Biosystems/Ambion, Austin, TX, USA). Next, 750 ng of the labeled cRNA were hybridized to Illumina HumanRef-8 v2 Expression BeadChips (Illumina Inc) overnight at 58°C. After washing, signals were developed with streptavidin-Cy3, and the BeadChips were scanned with the BeadArray Reader and processed using BeadStudio software obtained from Illumina.

### Microarray data sets and analyses

For the training cohort of 149 breast cancers, scanned Illumina microarray image data were extracted and processed by Gene Expression Module version 3.4 of BeadStudio software (Illumina Inc) using a background subtraction and a quantile normalization method for direct hybridization assays. Normalized hybridization intensity values were adjusted by assigning a constant value of 16 to any intensity value lower than 16, according to the recommendation by the MAQC Consortium [21]. A log_2 _expression ratio of an intensity value to the average signal value for each transcript in all samples was calculated. The training cohort microarray data are available at the Gene Expression Omnibus website [GSE:16987] [22].

An independent validation cohort consisting of publicly available gene expression array data from 2,487 breast cancers was compiled from different published original reference data sets that used Agilent and Affymetrix microarray platforms (Table S2 in Additional file 2). On the basis of the clinical treatment and the end point, we used four subgroups of the validation cohort to validate the CMTC classification derived from the training cohort: (1) 2,239 cancers with follow-up [23-36], (2) 1,058 cancers without adjuvant therapy [24-31,34], (3) 756 ER+ cancers with or without ET [24,26-29,33] and (4) 248 breast cancers treated with neoadjuvant chemotherapy and pCR information [37]. The methods of platform-specific data treatment and analyses are described in the Supplemental methods in Additional file 3.

## Results

### Gene model and generation of the ClinicoMolecular Triad Classification

Of the 149 evaluable breast cancers in the training cohort (Table S1 in Additional file 1), we grouped all 26 Her2+ tumors and 18 TN tumors into one group and the remaining 105 into another group in the first round of supervised clustering analysis to identify the differentially expressed genes. After two screens (see Supplemental methods in Additional file 3 and Figure S1 in Additional file 4), we obtained a molecular profile of Her2+/TN with 1,304 genes (1,349 oligonucleotide probes; some genes were represented by multiple oligonucleotide probes in the Illumina BeadChip. This molecular profile appeared to divide the 149 tumors into a familiar three-group pattern (Figure S1B in Additional file 4) in which the third group included most of the Her2+/TN tumors. Compared to the 16 published prognostic gene expression signatures (Table S3 in Additional file 5), a total of 501 genes were found in the list of the 1,304 genes matching 4% to 90.4% of the genes in these prognostic signatures. These overlapped genes included the following: (1) 29% (223 of 769) of the genes in the estrogen-regulated gene expression signature [38] and 14% (10 of 70) of the Rotterdam signature (76GS) [25]; (2) two ER-related gene signatures, 18% (92 of 512) of the intrinsic gene subtype signature (subtype) [6,7] and 56% (28 of 50) of the modified subtype classifier 50-gene prediction analysis of microarray (PAM50) [23]; (3) 10% (106 of 1,025) of the embryonic stem cell-like gene signature [39], 16% (29 of 181) of the "invasiveness" gene signature [40], 20% (32 of 155) of the stroma-derived prognostic predictor [41] and 14% (8 of 58) of the CD44 signature [42]; four stem cell-related gene signatures, 86% (93 of 108) of the Genomic Grade Index (97GS) [26], 90% (75 of 83) of the proliferation gene signature [31], 48% (11 of 23) of the *TP53 *mutation gene signature [29], 16% (73 of 462) of the wound-response gene signature (WS) [43], 30% of the lethal phenotype gene signature (37GS) [44]; and 42% (26 of 62) of MammaPrint (70GS) [24] and 56% (9 of 16) of Oncotype DX [45], with these latter two being the most widely used gene signatures [19].

**Figure 1:**
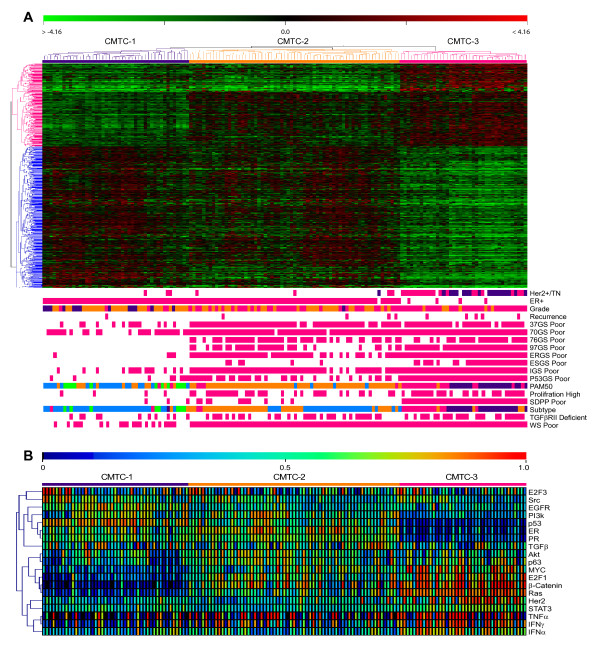
***CMTC *gene expression pattern, prognostic framework, and oncogenic pathway activity**. **(A) **The 803-gene signature (represented by 828 oligonucleotide probes) was used to classify the gene expression pattern in the 149 breast cancers in the training cohort into the three main clusters of CMTC. Tumors in CMTC-3 were mostly Her2+/TN as well as CMTC-1 and CMTC-2 non-Her2+/TN. The bottom multicolor bars indicating Her2+/TN are as follows: Her2+, deep pink; TN, dark blue. The multicolor bars indicating grade are as follows: grade 1, dark blue; grade 2, dark orange; and grade 3, deep pink. The multicolor bars indicating subtype and PAM50 are as follows: normal-like, lime; luminal A, blue; luminal B, dark orange; basal-like, dark blue; and Her2+, deep pink. CMTC = ClinicoMolecular Triad Classification; Her2 = human epidermal growth factor receptor 2; TN = triple-negative; ER2+ = estrogen receptor-positive; TGFβRII = transforming growth factor β receptor type II. **(B) **The probabilities of pathway activation of 19 published oncogenic pathway signatures in the 149 breast cancers in the training cohort. Blue indicates low pathway activity, and red indicates high activity. EGFR = epidermal growth factor receptor; PAM50 = 50-gene prediction analysis of microarray; PI3K = phosphatidylinositol 3-kinase; PR = progesterone receptor; STAT3 = signal transducer and activator of transcription 3.

To eliminate any potential confounding effects due to these prognostic signatures, we excluded all of the 501 overlapping genes from the list of 1,304 genes and used the remaining 803 genes (828 oligonucleotide probes) (Table S4 in Additional file 6) to perform a clustering analysis on the 149 tumors. The pattern with three main clusters was again apparent in the dendrogram (Figure 1A). The differential gene expression patterns were significantly different among the three groups as determined by performing an analysis of variance test (*P <*0.00001 among the three groups) and a *t*-test (corrected to *P <*0.01 between any two groups). We termed this 803-gene signature the "ClinicoMolecular Triad Classification," in which CMTC-3 contains most of the Her2+/TN tumors (92.3%). This 803-gene set was used as the new CMTC classifier for further analysis to categorize breast cancer in the validation cohort by a correlation method (Supplemental methods in Additional file 3).

### ClinicoMolecular Triad Classification correlates to clinical parameters of breast cancer

To understand the relationship between the gene expression profiles and the clinicopathological characteristics of CMTC, the three CMTC tumor types were compared based on their clinical and pathological parameters in 149 breast cancers in the training cohort and in 2,487 breast cancers in the validation cohort (Table 1). The latter cohort consisted of all evaluable breast cancers from published microarray data that had complete pathological and clinical outcome data. We found a statistically significant association between CMTC-3 tumors and larger size, high grade, low ER expression and mostly Her2+/TN phenotypes in both training and validation cohorts. In contrast, CMTC-1 tumors were smaller and low-grade, had high ER expression and were rarely the Her2+/TN phenotype. CMTC-2 tumors were larger in size and high-grade, had high ER expression and were rarely the Her2+/TN phenotype.

**Table 1:** Clinical and pathological variables in ClinicoMolecular Triad Classification of breast cancer in training and validation cohorts

	Training cohort (*n *= 149)				Validation cohort (*n *= 2,487)			
	
Variables	CMTC-1, *n *(%)	CMTC-2, *n *(%)	CMTC-3, *n *(%)	*P *value	CMTC-1, *n *(%)	CMTC-2, *n *(%)	CMTC-3, *n *(%)	*P *value
Total	45 (30.2)	65 (43.6)	39 (26.2)		803 (32.3)	794 (31.9)	890 (35.8)	
Age								
< 50	15 (33.3)	18 (27.7)	17 (43.6)	2.51E-01	231 (39.1)	202 (34.9)	299 (43.6)	6.30E-03
≥ 50	30 (66.7)	47 (72.3)	22 (56.4)		360 (60.9)	377 (65.1)	386 (56.4)	
Size								
≤ 2 cm	23 (51.1)	21 (32.3)	11 (28.2)	5.62E-02	361 (54.7)	209 (32.5)	235 (32.4)	1.05E-20
> 2 cm	22 (48.9)	44 (67.7)	28 (71.8)		299 (45.3)	434 (67.5)	490 (67.6)	
LN-	26 (59.1)	21 (32.3)	24 (61.5)	3.27E-03	490 (66.8)	436 (59.2)	498 (60.3)	4.37E-03
LN+	18 (40.9)	44 (67.7)	15 (38.5)		243 (33.2)	301 (40.8)	328 (39.7)	
Grade								
1	13 (28.9)	1 (1.5)	0 (0.0)	5.55E-13	270 (39.4)	81 (12.2)	29 (3.9)	3.47E-130
2	27 (60.0)	30 (46.2)	6 (15.4)		342 (49.9)	339 (51.2)	220 (29.6)	
3	5 (11.1)	34 (52.3)	33 (84.6)		74 (10.8)	242 (36.6)	495 (66.5)	
ER-	0 (0.0)	1 (1.5)	35 (89.7)	1.16E-27	69 (8.6)	45 (5.7)	584 (65.6)	2.60E-211
ER+	45 (100)	64 (98.5)	4 (10.3)		734 (91.4)	749 (94.3)	306 (34.4)	
Her2+/TN								
No	42 (93.3)	60 (92.3)	3 (7.7)	1.87E-22	715 (89.0)	668 (84.1)	238 (26.7)	1.45E-197
Yes	3 (6.7)	5 (7.7)	36 (92.3)		88 (11.0)	126 (15.9)	652 (73.3)	
Recurrence								
No	44 (97.8)	61 (93.8)	34 (87.2)	1.49E-01	595 (81.4)	423 (59.5)	486 (61.0)	1.99E-22
Yes	1 (2.2)	4 (6.2)	5 (12.8)		136 (18.6)	288 (40.5)	311 (39.0)	

CMTC = ClinicoMolecular Triad Classification; LN = lymph node status; ER = estrogen receptor; TN = triple-negative.

### ClinicoMolecular Triad Classification displays unique patterns in oncogenic signaling pathways

To understand the biological processes underlying our CMTC classification scheme, we compared the three CMTC groups in 149 breast cancers in the training cohort with 19 published microarray-based signaling pathway signatures [18,46] (Figure 1B). We found the highest activity in oncogenic signaling pathways involving Her2, Myc, E2F1, β-catenin and Ras in CMTC-3 and a negative correlation with the activities of ER, PR and p53 wild-type pathways. In contrast, CMTC-1 tumors demonstrated low activity in Myc, E2F1, β-catenin, Ras, IFN-γ and Her2 signaling pathways and higher activity in ER, PR and p53 wild-type pathways. CMTC-2 was distinct from the other two groups in having high activities in most of the oncogenic pathways that differentiated CMTC-1 from CMTC-3, including the ER, phosphatidylinositol 3-kinase (PI3K), Myc and β-catenin pathways.

### ClinicoMolecular Triad Classification unifies prognostication from published prognostic gene signatures

Of the 16 published prognostic gene signatures (Table S3 in Additional file 5), 14 microarray-based signatures were used as risk classifiers to evaluate the 149 breast cancers in the training cohort. Even when all the overlapping genes from these published prognostic gene signatures were excluded from the CMTC classifier gene set, the tumors classified as carrying a "poor prognosis" according to the published prognostic gene signatures were mostly found in CMTC-3 and CMTC-2 and infrequently in CMTC-1 (Figure 1A). Comparison of the five molecular subtypes [6,7] revealed that all the normal-like tumors were found in CMTC-1, luminal A tumors were distributed in both CMTC1 and CMTC-2, luminal B tumors were mainly found in CMTC-2 and almost all Her2+ and basal-like subtypes were found CMTC-3. A similar distribution of the five molecular subtypes was also observed when we used a newer intrinsic subtype classifier, PAM50 [23], a 50-gene subtype predictor, with more luminal B tumors grouped into CMTC-2 (Figure 1A).

### ClinicoMolecular Triad Classification correlates with clinical outcomes in breast cancer

**Figure 2:**
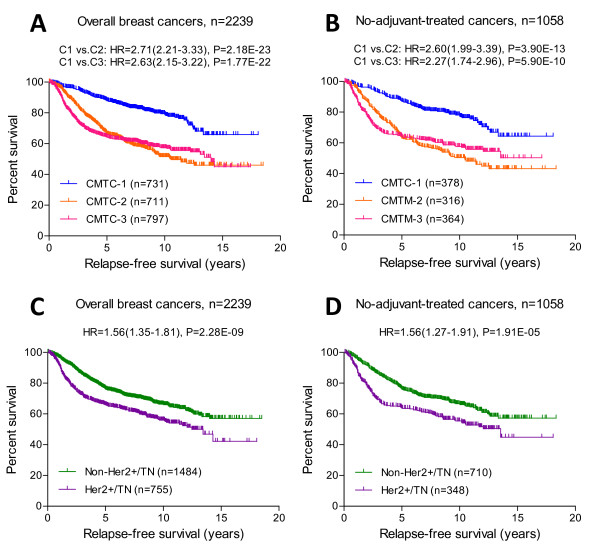
***CMTC *and Her2+/TN status in prediction of the clinical outcomes**. Kaplan-Meier analysis was used to compare relapse-free patient survival among the CMTC-1 (C1), CMTC-2 (C2) and CMTC-3 (C3) in **(A) **2,239 breast cancers overall and **(B) **1,058 nonadjuvant treatment cancers, as well as in **(C) **Her2+/TN and non-Her2+/TN 2,239 breast cancers overall and **(D) **1,058 nonadjuvant treatment cancers. The hazard ratios with 95% confidence intervals in parentheses were calculated using the Cox proportional hazards method. The *P *values were determined using the log-rank test. CMTC = ClinicoMolecular Triad Classification; Her2 = human epidermal growth factor receptor 2; HR = hazard ratio; TN = triple-negative.

During our first clinical follow-up (mean follow-up = 31 months) for the 149 cancers in the training cohort, five recurrences (5 of 39 = 12.8%) were found in CMTC-3, four (4 of 65 = 6.2%) were found in CMTC-2 and only one (1 of 45 = 2.2%) was found in CMTC-1. However, these results were not statistically significant, owing to a low event rate in a short follow-up period (Figure 1A and Table 1). In the validation breast cancer cohort with long-term follow-up, a significantly higher recurrence rate was observed: 40.5% in CMTC-2 and 39% in CMTC-3 compared to 18.6% in CMTC-1 (Table 1). The Kaplan-Meier analyses for relapse-free survival showed significant differences between CMTC-1 and CMTC-2 and also between CMTC-1 and CMTC-3 breast cancer patients in 2,239 breast cancers overall (Figure 2A) and in 1,058 breast cancers in which the patients in the validation cohort did not receive any adjuvant therapy (Figure 2B). CMTC-2 and CMTC-3 patients had similar poor prognoses (Figure 2A and 2B). By using a Cox proportional hazards model (Table S5 in Additional file 7), we compared CMTC-2 and CMTC-3 to CMTC-1 and found that, on the basis of univariate analysis, the hazard ratio (HR) was the highest among all clinicopathological parameters and prognostic signatures (HR = 2.40, 95% confidence interval (95% CI) = 1.88 to 3.05; *P <*0.01). By using multivariate analysis, we again found that CMTC had the highest HR (HR = 1.73, 95% CI = 1.23 to 2.44; *P <*0.01) among all clinicopathological parameters (age, nodal status, tumor size, tumor grade and receptor status). Among all the prognostic gene signatures, the HR of CMTC was the highest in univariate analysis (HR = 2.40, 95% CI = 1.88 to 3.05; *P <*0.0001) and the second highest in multivariate analysis (HR = 1.43, 95% CI = 1.00 to 2.04; *P <*0.05). Prediction of recurrence using CMTC was also better than that using receptor status Her2+/TN (Her2+/TN vs non-Her2+/TN) (Figures 2C and 2D). Her2+/TN receptor status had a HR of 1.56 in univariate analysis (95% CI = 1.27 to 1.91; *P <*0.01) and 1.35 in multivariate analysis (95% CI = 0.91 to 2.00; *P *= 0.13), suggesting that CMTC was more robust than receptor status alone in predicting survival. Hence, CMTC is an independent, strong predictor of recurrence in breast cancer.

### ClinicoMolecular Triad Classification correlates with the benefits of endocrine therapy

**Figure 3:**
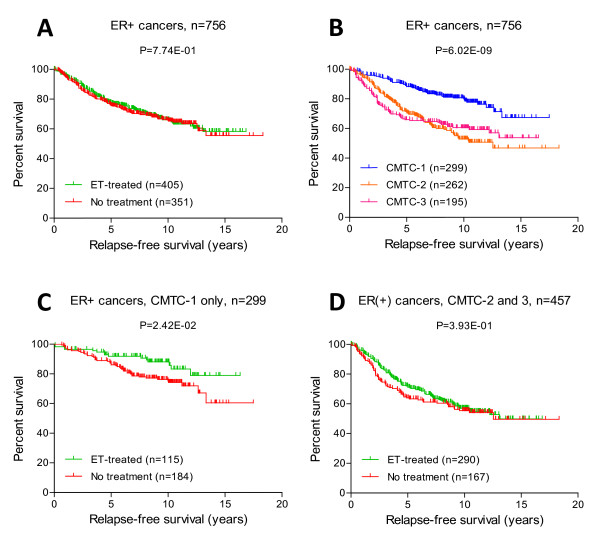
***CMTC *and the prediction of benefits of ET in ER+ breast cancer**. Kaplan-Meier analysis was used to compare patients' relapse-free survival **(A) **between ET treatment and no treatment **(B) **among all 756 ER+ breast cancers, **(C) **between the three CMTC groups of all 756 ER+ breast cancers and ET treatment vs no treatment in 299 CMTC-1-only ER+ cancers and **(D) **and ET treatment vs no treatment between 457 CMTC-2- and CMTC-3-only ER+ cancers. The *P *values were determined using the log-rank test. CMTC = ClinicoMolecular Triad Classification; ER = estrogen receptor; ET = endocrine therapy; TN = triple-negative.

In the validation cohort, from among the group of 756 patients with ER+ breast cancer, 405 received ET (390 patients received tamoxifen and 15 patients received an unspecified hormonal therapy) and the remaining 351 did not receive any adjuvant therapy. These two groups were not matched, as they were not derived from a randomized, controlled trial. To identify the association between CMTC and tumor response to ET, we compared the relapse-free survival rates between the two groups. Interestingly, we did not see any benefit of ET (*P *= 0.7735) when we compared the treated and untreated groups in the entire 756 ER+ breast cancer population (Figure 3A). However, when we divided the 756 ER+ patients into the three CMTC groups, patients in CMTC-1 group had good clinical outcomes in general (Figure 3B), particularly in the 115 patients treated with ET compared to the 184 untreated patients (Figure 3C). In fact, the benefit of ET was observed only in the CMTC-1 patients (Figure 3C) and not in the CMTC-2 and CMTC-3 patients (Figure 3D). Hence, in our validation cohort, CMTC appeared to predict a benefit from ET in ER+ breast cancer. The other gene signatures could demonstrate only varying degrees of prognostic significance, but did not predict the benefit of ET in the 756 ER+ breast cancer patients (Table S6 in Additional file 8). When we tried to stratify the patients into different cancer stages, only a limited number of cases in the validation cohort had complete staging information. On the basis of all the data available, we observed only a trend toward better relapse-free survival associated with ET in treated versus untreated ER+, CMTC-1 patients at stage I (*n *= 155; *P *= 0.0967) and at stage II or worse (*n *= 142; *P *= 0.0612) (Figure S2 in Additional file 9).

### ClinicoMolecular Triad Classification predicts complete pathological response to neoadjuvant chemotherapy

**Figure 4:**
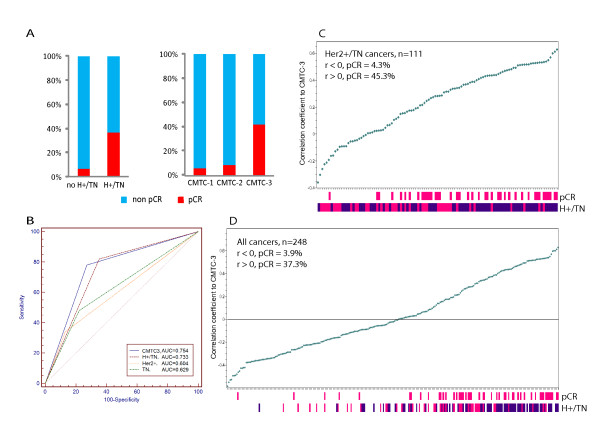
***CMTC *and prediction in pCR of neoadjuvant chemotherapy**. **(A) **The percentage of pCR between non-Her2+/TN tumors (non-H+/TN) and Her2+/TN tumors (H+/TN) and within the three CMTC groups of the 248 breast cancers with neoadjuvant chemotherapy. **(B) **Comparison of area under the curve (AUC) to predict pCR in CMTC-3 tumors (CMTC-3 vs CMTC-1 and CMTC-2; *P *= 0.0001), Her2+/TN tumors (Her2+/TN vs non-Her2+/TN; *P *= 0.0001), Her2+ tumors (Her2+ vs Her2-; *P *= 0.0245) and TN tumors (TN vs non-TN; *P *= 0.0052). By comparing the gene profiles of individual tumors with CMTC-3, a correlation coefficient (*r*) was calculated as an index reflecting its degree of similarity to the expression pattern of CMTC-3 tumors. The two graphs show the relationship between *r *value and pCR **(C) **in the 111 Her2+/TN cancers and **(D) **in all 248 cancers. pCR status (deep pink), Her2+ status (deep pink) and TN status (dark blue), respectively, are indicated by the bottom bars. AUC = area under the curve; CMTC = ClinicoMolecular Triad Classification; Her2 = human epidermal growth factor receptor 2; pCR = pathological response; TN = triple-negative.

To determine whether CMTC could predict tumor responses to neoadjuvant chemotherapy, 248 breast cancer patients [37] from the validation cohort who received neoadjuvant chemotherapy were studied to determine the relationship between CMTC groups and complete pCR. The highest pCR rate was found in CMTC-3 breast cancer (42%), with much lower pCR rates in CMTC-1 breast cancer (6%) and CMTC-2 breast cancer (8%). Her2+/TN breast cancer patients had a 37% pCR rate (Figure 4A). To compare the relative ability of receptor status (Figure 4B) and gene signature (Table S7 in Additional file 10) to predict pCR, we calculated the area under the curve (AUC) using receiver operating characteristic (ROC) curve analyses. We found that CMTC-3 tumors had the highest AUC value (0.754) compared to Her2+/TN tumors (0.733), Her2+ tumors (0.604) and TN tumors (0.629) (Figure 4B). In addition, tumors with a high positive correlation with CMTC-3 were significantly correlated with pCR in 111 Her2+/TN tumors (Figure 4C) and in all 248 chemotherapy-treated tumors (Figure 4D). When we compared CMTC to 14 published prognostic gene signatures, the highest AUC values were found in the CMTC-3 group in all 248 cancers (0.811) (95% CI = 0.76 to 0.86; *P <*0.001) and in 111 Her2+/TN tumors (0.718) (95% CI = 0.63 to 0.80; *P <*0.001). CMTC was also better than the five intrinsic subtypes and PAM50, as well as the other gene signatures, in predicting pCR (Table S7 in Additional file 10). For comparison purposes, we also tabulated the sensitivity, specificity, positive predictive value (PPV), negative predictive value (NPV) and accuracy of CMTC in predicting pCR together with the other gene signatures (Table S8 in Additional file 11). Again, CMTC remained one of the best predictors among these gene signatures, with a good balance between sensitivity and specificity.

## Discussion

Using the gene signature generated from the training cohort, we identified an expression pattern of 1,304 genes that divided the 149 breast cancers into three distinct groups, in which Her2+/TN breast cancer represented 90.4% of the 39 group 3 tumors (Figure S1B in Additional file 4). Of the 1,304 genes, a total of 501 genes overlapped with 16 published prognostic gene signatures (Table S3 in Additional file 5), matching 4% to 90.4% of the genes in these gene signatures. The high rate of the overlapped genes across the different published gene signatures suggests strong clinical and biological relevance.

To remove any potential confounding effects of the overlapping genes from these published gene signatures, we excluded all of the 501 genes in these published gene signatures that overlapped with our original 1,304-gene set. As a result, a unique 803-gene set (represented by 828 oligonucleotide probes in the Illumina BeadChip assay) was derived. Using the new probe set, we observed a dendrogram with three main clusters which we have termed the "ClinicoMolecular Triad Classification." In the CMTC, the gene expression pattern of CMTC-1 is completely opposite that of CMTC-3 and results in a distinct, intermediate CMTC-2 (Figure 1A). The tumors in CMTC-1 and CMTC-2 were mostly ER+ and rarely Her2+/TN. However, of the 44 Her2+/TN tumors, 36 (81.82%) were found in CMTC-3. When we applied the CMTC to 866 Her2+/TN tumors in the 2,487 validation breast cancers, 652 (75.3%) were assigned to the CMTC-3 group (Table 1). Furthermore, the prognostic predictability of CMTC agreed very well with all 14 prognostic gene signatures that were developed independently using different commercial microarray platforms (Table S3 in Additional file 5). Using these prognostic gene signatures, we found tumors carrying a poor prognosis (from signatures dichotomized into good vs poor prognosis) mostly in the CMTC-2 and CMTC-3 cohorts (Figure 1A). There was also a close correlation between the five molecular subtypes [6,7,23].

In both training and validation cohorts, the tumors in CMTC-1 were of smaller size and lower grade than tumors in the CMTC-2 and CMTC-3 groups. In the validation cohort, patients in the CMTC-1 cohort were found to have significantly better clinical outcomes than the patients in the CMTC-2 and CMTC-3 groups as demonstrated in 2,239 breast cancers overall (Figure 2A), 1,058 non-adjuvant-treated cancers (Figure 2B) and 756 ER+ cancers (Figure 3B). CMTC was better at predicting clinical outcomes than receptor status alone (Figure 2C and 2D), suggesting that it reflects not only the presence of the receptors but also pathway activity. Furthermore, on the basis of the survival data of 1,058 breast cancer patients from the validation cohort who did not receive adjuvant therapy, CMTC prognosticated clinical outcomes significantly better than other published gene signature predictors (Table S5 in Additional file 7).

Another potential application of our molecular classification is in the prediction of response to adjuvant ET and neoadjuvant chemotherapy. Because of the limitation of using public microarray databases as our validation cohort, we are not able to conclude that CMTC can predict treatment response [47,48]. We were not able to match the treatment arms according to CMTC groups, as they were not randomized as such. Therefore, our intent in this study was to demonstrate an association between CMTC and tumor response to a specific treatment modality by treating each breast cancer case in our validation cohort as a randomly selected patient. CMTC-1 patients appeared to benefit the most from ET in terms of recurrence-free survival compared to patients with ER+ breast cancer who did not receive ET (Figure 3C), but the benefits of ET were not significant in CMTC-2 and CMTC-3 patients (Figure 3D). Using the same validation cohort, we found that CMTC also appeared to be better than other published prognostic gene signatures in predicting responses to ET (Table S6 in Additional file 8). Figure 3A shows that the benefit of ET was nullified by the fact that most of the ET-treated breast cancers were classified as CMTC-2 and CMTC-3 (*n *= 290) (Figure 3D) rather than CMTC-1 (*n *= 115) (Figure 3C). Conversely, most of the group that received no treatment were classified as CMTC-1. Furthermore, it may be possible that ET-treated patients presented at a later stage of their disease than did those who received no treatment, given that the breast cancers classified as CMTC-2 and CMTC-3 were associated with larger tumor size (see preceding paragraph). However, subgroup analyses failed to reach statistical significance, as many cases in the validation cohort lacked complete staging information. On the basis of all the data available, we did detect a trend toward better relapse-free survival in both stage I (*n *= 155; *P *= 0.0967) and stage II or worse (*n *= 142; *P *= 0.0612) CMTC-1 ER+ ET-treated patients (Figure S2 in Additional file 9). Therefore, in our validation cohort, there was more ET given to so-called "nonresponders" than to "responders." This brings up an important point: If we do not have a better way to classify ER+ breast cancer and use ET to treat all ER+ breast cancers equally, we may not achieve the desired clinical benefit. This result will need to be confirmed in a randomized, controlled trial with a larger set of ER+ patients and complete staging information.

With regard to response to neoadjuvant chemotherapy, CMTC-3 tumors demonstrated a higher rate of complete pCR to neoadjuvant chemotherapy than the other two CMTC groups did (Figure 4A). The ability of CMTC to predict pCR after neoadjuvant chemotherapy is not only superior to receptor status (Her2+, TN and Her2+/TN) (Figure 4B) but also better than the other independent prognostic gene signatures (Table S7 in Additional file 10). Several gene signatures have been reported to predict pCR or clinical response to specific types of chemotherapy in relatively few, highly selected patients (see Table 1 in [49]). Interestingly, the NPV, PPV and accuracy of these chemotherapy-specific predictors are all within a range similar to that of CMTC, except that CMTC is applicable to different chemotherapeutic regimens in all breast cancers and is prognostic in addition to its predictive power for pCR.

To examine the biological processes that may be involved in CMTC, oncogenic signaling pathway analyses were performed in the training cohort, which showed that CMTC-3 tumors had the highest activity in Her2 and other oncogenic signaling pathways (Myc, E2F1, β-catenin, Ras and IFN-γ) and the lowest activity in ER, PR and wild-type p53 pathways (Figure 1B). This oncogenic pathway pattern was completely opposite to that of CMTC-1 tumors. CMTC-2 was distinct from the other two groups in having high activity in most of the oncogenic pathways that differentiated CMTC-1 from CMTC-3. Unlike CMTC-1 and CMTC-3 tumors, CMTC-2 tumors did not respond well to the two common treatment strategies, namely, ET and chemotherapy. To find new molecular targets for CMTC-2 tumors, our next study will focus on the molecular profiles of CMTC-2 tumors to identify novel treatment strategies. For example, most CMTC-2 tumors displayed activity in the PI3K and β-catenin pathways, and patients with these tumors may benefit from targeted therapies that disrupt these pathways and ER blockage.

The microarray data of our training cohort were generated predominantly from FNABs taken from an unselected cohort of clinical patients prior to any surgical or medical interventions. Thus, CMTC could be used to help in making treatment decisions at the point of diagnosis. Since CMTC can predict treatment outcomes better than standard surgical pathological parameters, FNABs taken for CMTC group assignment of breast cancer patients in the future may help clinicians decide which patients will benefit from neoadjuvant chemotherapy. Another advantage of using FNABs in our study was the ability to include smaller tumors, which are becoming more common in the era of screening mammography but are routinely excluded from tissue banking because of size limitations, an issue shared by most reported microarray-based prognostic gene signatures. FNABs appeared to collect malignant epithelial cells selectively, as demonstrated by over 80% of malignant cells found in our FNAB specimens. Our microarray data were also very reproducible in duplicate specimens (*R *= 0.9918) (Supplemental methods in Additional file 3).

The gene profiles used to develop CMTC were derived from a commercially available whole-genome microarray platform that has become more affordable than currently available multigene assays, such as MammaPrint (70GS; Agendia Inc, Irvine, CA, USA) and Oncotype DX (Genomic Health, Redwood City, CA, USA), which report only a limited number of genes [24,45] at a high cost [19,50]. Furthermore, the clinical application of CMTC may be extended to other commercial genome-wide microarray platforms, as we have demonstrated the reproducibility of CMTC classification in the validation cohort derived independently from different DNA microarray platforms. Another potential application of using a whole-genome microarray platform is the ability to perform pathway activity analyses to provide insights into the biological processes operating within the breast cancer, and this may help to identify novel treatment strategies.

During the past decade, the focus of research has been on finding a gene signature that is both prognostic and predictive with high accuracy while containing only a small number of genes. However, with better microarray technology available at a lower price, we are able to generate microarray data that is highly reproducible and cheaper than any of the commercially available gene signatures. It is well known that single-gene estimation (for example, ER) of individual pathway activity is not accurate enough to predict treatment outcomes (for example, response to ET). Therefore, we believe that by using a larger number of genes, the test will be less susceptible to variations caused by errors in measuring individual genes and thus will result in a more reliable determination of the activity levels of critical oncogenic pathways involved in prognosis and treatment response. With the current vastly improved computing power and storage capacity, we advocate using genome-wide gene profiles to provide a more comprehensive genomic analysis comprising a portfolio of current gene expression profiles that includes CMTC, complete oncogenic pathway analyses and the potential for future analyses if pathway gene signatures are further refined.

Finally, CMTC will need to be validated by prospective, randomized, clinical studies, which are in our future plans. On the basis of our present study, we can say that CMTC has the potential to guide treatment decisions at the time of diagnosis, such as the consideration of treating CMTC-3 breast cancer with neoadjuvant chemotherapy, CMTC-1 with ET alone and CMTC-2 with a combination of ET and chemotherapy in adjuvant settings. We note that CMTC-2 remains a challenge in terms of finding an effective treatment. Additional targeted therapies are necessary, and our oncogenic pathway analyses may provide some guidance in finding targets for CMTC-2.

## Conclusions

On the basis of the Her2+/TN molecular phenotype, we developed an 803-gene signature, the ClinicoMolecular Triad Classification system, which is a new, clinically useful molecular classification scheme for breast cancer. Similarly to current clinical practice, CMTC divides breast cancer into three distinct groups. Patients assigned to CMTC-1 have a better prognosis and significantly benefit from ET. Patients in categories CMTC-2 and CMTC-3 have worse clinical outcomes than CMTC-1 patients, with CMTC-3 tumors tending to display a higher rate of complete pCR in response to neoadjuvant chemotherapies. On the basis of our validation analyses using all evaluable public microarray data, the benefits of our clinicomolecular grouping include (1) the capacity to determine the patient's CMTC group preoperatively, which is especially important in neoadjuvant settings; (2) a further improvement in the ability to predict clinical outcomes and treatment responses to ET and neoadjuvant chemotherapy over clinical receptor status and currently available gene signatures; (3) a molecular classification system that is more generalizable than other prognostic gene signatures (including ER+, ER-, tumors of any size, node-positive or node-negative breast cancer) and was reproducible in the validation cohort, from which the data were generated using different commercially available microarray platforms; and (4) the potential to identify novel molecular targets for each CMTC breast cancer group, especially for CMTC-2 tumors that do not respond well to either ET or chemotherapy. Once we have validated the CMTC system in prospective clinical trials, we plan to introduce it into the clinic to help physicians guide treatment decision-making.

## Abbreviations

AUC: area under the curve; CMTC: ClinicoMolecular Triad Classification; ER: estrogen receptor; ET: endocrine therapy; FNAB: fine-needle aspiration biopsy; Her2: human epidermal growth factor receptor 2 (also known as *ERBB2*); IFN: interferon; NPV: negative predictive value; pCR: pathological response; PI3K: phosphatidylinositol 3-knase; PPV: positive predictive value; PR: progesterone receptor; RIN: RNA integrity number; ROC: receiver operating characteristic analysis; RT-PCR: reverse transcriptase polymerase chain reaction; TN: triple-negative (ER-/PR-/Her2-).

## Competing interests

The authors declare that they have no competing interests.

## Authors' contributions

DYW and WLL designed the project and analyzed the data. DRM, SK and WLL contributed to the collection of clinical material. SJD and SB are associated pathologists. The manuscript was prepared by DYW and WLL and modified by SJD and DRM. All authors read and approved the final manuscript.

## Supplementary Material

Additional file 1 **Supplementary Table S1 Summary of patient information and tumor pathological data for the training cohort of 149 breast cancers**. CMTC = ClinicoMolecular Triad Classification; EIC = extensive intraductal component; IDC = invasive ductal carcinoma; LVI = lymphovascular invasion; PTID = Patient's identity number; RIN = RNA integrity number. Click here for file

Additional file 2 **Supplementary Table S2 Summary of resource, platform, adjuvant treatment status and clinical end point of the microarray data sets used in this study**. DMFS = distant metastasis-free survival; RFS = relapse-free survival. Click here for file

Additional file 3 **Supplementary methods**. File containing descriptions of microarray data resources, platform-specific data treatment and analyses, integration of published gene expression signatures and signaling pathway signatures, and generation of gene expression profiles for the Her2+/TN phenotype. ANOVA = analysis of variance; ER = estrogen receptor; *ESR1 *= estrogen receptor 1 gene; GEO = Gene Expression Omnibus; Her2 = human epidermal growth factor receptor 2; MAQC = MicroArray Quality Control project; PAM50 = 50-gene prediction analysis of microarray; PM-MM = Pairs of Perfect Match(PM) and Mismatch(MM) oligonucleotide probes; PR = progesterone receptor; TN = triple-negative; WS = wound-response gene signature. Click here for file

Additional file 4 **Supplementary Figure S1 Generation of gene expression profile for Her2+/TN phenotype in the training cohort (*n *= 149)**. **(A) **First screening of Her2+/TN-related genes. A group of 44 Her2+/TN breast cancers were used to distinguish the gene expression from the other 105 tumors. A total of 1,428 probes were selected at a level of the Bonferroni-corrected *P *value < 0.01. By using the 1,428-probe set in a hierarchical clustering pattern, 39 tumors that were mostly Her2+/TN formed group 3, with two other subgroups emerging on the heat map: groups 1 and 2. **(B) **Second screening for the most differentially expressed genes between the three groups. By performing an analysis of variance test, 1,349 probes were selected at a level of *P *< 0.001 among the three groups. A three-cluster pattern is apparent on the heat map, based on hierarchical clustering analysis using the 1,349-probe set. The tumors with Her2+/TN status were 2.4% (1 of 42) in group 1, 10.3% (7 of 68) in group 2 and 92.3% (36 of 39) in group 3. The bottom color bars represent Her2+ (deep pink) and TN (blue). ANOVA = analysis of variance; Her2 = human epidermal growth factor receptor 2; TN = triple-negative. Click here for file

Additional file 5 **Supplementary Table S3 Summary of name, definition, platform and reference of the prognostic signatures used in this study and the overlapped genes between ClinicoMolecular Triad Classification and published independent breast cancer gene expression prognostic signatures**. TGF = transforming growth factor. Click here for file

Additional file 6 **Supplementary Table S4 CMTC 828-probe set including Illumina probe ID, gene symbol and expression log_2 _ratio for each of the 828 probes, as well as the mean log_2 _ratios and statistical relationships among the three CMTC groups of 149 breast cancers in the training cohort**. CMTC = ClinicoMolecular Triad Classification. Click here for file

Additional file 7 **Supplementary Table S5 Univariate and multivariate analyses of standard clinicopathological parameters, 14 independent gene signatures and CMTC as prognostic indicators for relapse among 1,058 breast cancer patients without adjuvant therapy in the validation cohort**. CI = confidence interval; CMTC = ClinicoMolecular Triad Classification; ER = estrogen receptor; ERGS = estrogen-regulated gene expression signature; ESGS = embryonic stem cell-like gene signature; Her2 = human epidermal growth factor receptor 2; IGS = "invasiveness" gene signature; LN = lymph node status; PAM50 = 50-gene prediction analysis of microarray; SDPP = stroma-derived prognostic predictor; TGFβRII = transforming growth factor β receptor type II; TN = triple-negative; WS = wound-response gene signature. Click here for file

Additional file 8 **Supplementary Table S6 Association between relapse-free survivals and Her2+/TN status**. Fourteen gene signatures and CMTC in the seven hundred fifty-six ER+ breast cancer patients with or without ET. ER = estrogen receptor; ERGS = estrogen-regulated gene expression signature; ESGS = embryonic stem cell-like gene signature; PAM50 = 50-gene prediction analysis of microarray; SDPP = stroma-derived prognostic predictor; TGFβRII = transforming growth factor β receptor type II; TN = triple-negative; WS = wound-response gene signature. Click here for file

Additional file 9 **Supplementary Figure S2 Benefits of ET in CMTC-1 ER+ breast cancer at different cancer stages**. Kaplan-Meier analyses were used to compare relapse-free survival between ET-treated and no-treatment ER+ breast cancer **(A) **in 155 stage I CMTC-1 cancers and **(B) **in 142 stage II or worse (stage II+) CMTC-1 cancers. The *P *values were determined by using the log-rank test. CMTC = ClinicoMolecular Triad Classification; ER = estrogen receptor; ET = endocrine therapy. Click here for file

Additional file 10 **Supplementary Table S7 Receiver operating characteristic analysis of the ability of independent gene expression signatures to predict pathological complete responses in breast cancer treated with neoadjuvant chemotherapy**. CI = confidence interval; CMTC = ClinicoMolecular Triad Classification; ERGS = estrogen-regulated gene expression signature; ESGS = embryonic stem cell-like gene signature; Her2 = human epidermal growth factor receptor 2; IGS = "invasiveness" gene signature; LumA = luminal A; LumB = luminal B; PAM50 = 50-gene prediction analysis of microarray; SDPP = stroma-derived prognostic predictor; TGFβRII = transforming growth factor β receptor type II; TN = triple-negative; WS = wound-response gene signature. Click here for file

Additional file 11 **Supplemental Table S8 The prediction of pCRs in 248 breast cancer patients treated with neoadjuvant chemotherapy on the basis of CMTC and 14 independent prognostic gene expression signatures**. CMTC = ClinicoMolecular Triad Classification; PAM50 = 50-gene prediction analysis of microarray; SDPP = stroma-derived prognostic predictor; TGFβRII = transforming growth factor β receptor type II; WS = wound-response gene signature. Click here for file
