# Supplementary material for: A new gene expression signature, the ClinicoMolecular Triad Classification, may improve prediction and prognostication of breast cancer at the time of diagnosis
Source: Breast Cancer Res. 2011 Sep 22;13(5):R92. doi: 10.1186/bcr3017 (PMC3262204; doi:10.1186/bcr3017)
Supplement: Additional file 1 — Supplementary Table S1 Summary of patient information and tumor pathological data for the training cohort of 149 breast cancers. CMTC = ClinicoMolecular Triad Classification; EIC = extensive intraductal component; IDC = invasive ductal carcinoma; LVI = lymphovascular invasion; PTID = Patient's identity number; RIN = RNA integrity number. [file bcr3017-S1.PDF]

Table S1 Patient information and tumor pathological data for the training cohort of 149 breast cancers

PTID	RIN	Age	Tumor Type	Tumor Size (cm)	Tumor Grade	Positive nodes	LVI	EIC	ER	PR	Her2	Triple-	Recurrence	Follow-up (months)	CMTC Type
GP001	6.2	42	IDC	1.5	2	0(15)	(-)	(-)	(+)	(-)	(-)	No	n	44.43	3
GP002	8.7	56	IDC/Lobular	2.2	3	0(3)	(-)	(-)	(-)	(-)	(-)	Yes	n	39.47	2
GP003	7.7	40	IDC	1.5	2	0(7)	(-)	(-)	(+)	(+)	(-)	No	n	32.77	1
GP004	7.0	46	IDC	2.6	2	0(5)	(-)	(-)	(+)	(+)	(-)	No	n	46.43	1
GP006	7.3	63	IDC	1.8	1	0(3)	(-)	(-)	(+)	(-)	(-)	No	n	46.00	1
GP007	8.4	47	IDC	4	3	8(18)	(+)	(+)	(-)	(-)	(+)	No	n	39.30	3
GP008	8.7	48	IDC	1.9	2	2(11)	(-)	(-)	(+)	(+)	(-)	No	n	46.20	2
GP009	7.1	51	IDC	2.7	3	2(20)	(+)	(-)	(+)	(-)	(-)	No	n	45.73	2
GP010	7.2	72	IDC	3	3	0(1)	(-)	(-)	(+)	(+)	(-)	No	y	13.60	2
GP011	7.2	84	IDC	2.1	1	0(1)	(+)	(-)	(+)	(+)	(-)	No	n	43.00	1
GP012	7.4	72	IDC	1.5	1	0(2)	(-)	(-)	(+)	(+)	(-)	No	n	43.73	1
GP013	8.2	58	IDC	3.5	2	1(17)	(-)	(-)	(+)	(-)	(-)	No	n	48.33	3
GP014	7.6	49	IDC	3.6	2	0(4)	(-)	(+)	(+)	(+)	(-)	No	n	43.73	1
GP015	8.3	43	IDC	2.9	3	1(4)	(+)	(-)	(-)	(-)	(-)	Yes	n	32.30	3
GP016	8.1	73	IDC	2.8	3	2(20)	(-)	(-)	(+)	(-)	(-)	No	n	23.63	2
GP017	7.5	31	IDC	3.5	3	7(16)	(+)	(-)	(+)	(-)	(+)	No	n	43.70	3
GP018	8.7	67	IDC	2	2	1(19)	(+)	(-)	(+)	(+)	(-)	No	n	43.63	2
GP019	9.1	45	IDC	2.8	3	0(3)	(-)	(-)	(-)	(-)	(-)	Yes	n	22.77	3
GP020	9.0	46	IDC	2.8	3	0(3)	(-)	(-)	(-)	(-)	(-)	Yes	NA	NA	3
GP021	9.1	46	IDC	0.8	1	0(3)	(-)	(+)	(+)	(+)	(-)	No	n	36.90	1
GP022	9.0	68	IDC/Papilloma	1.4	2	0(2)	(-)	(-)	(+)	(+)	(-)	No	n	25.30	1
GP023	8.7	51	IDC	1.4	1	0(2)	(-)	(-)	(+)	(+)	(-)	No	n	35.97	1
GP024	8.1	80	IDC	2	3	0(1)	(-)	(-)	(-)	(-)	(-)	Yes	y	23.13	3
GP025	9.4	46	IDC	2	2	0(2)	(-)	(+)	(+)	(+)	(+)	No	n	21.63	1
GP026	8.3	48	IDC/lobular	2.1	2	0(1)	(-)	(-)	(+)	(+)	(-)	No	n	24.97	1
GP027	7.8	69	IDC	3.3	1	4(23)	(-)	(-)	(+)	(+)	(-)	No	n	36.33	1
GP029	6.8	45	IDC/lobular	4.2	3	1(25)	(+)	(-)	(+)	(+)	(-)	No	n	29.90	1
GP030	7.3	52	IDC	2.8	2	0(1)	(+)	(-)	(+)	(-)	(-)	No	n	38.80	2
GP031	8.6	29	IDC	1.9	3	0(4)	(-)	(-)	(-)	(-)	(-)	Yes	n	23.30	3
GP032	6.2	44	IDC	2.3	2	1(16)	(+)	(-)	(+)	(-)	(-)	No	n	38.83	2
GP033	8.4	56	IDC	2.5	3	13(28)	(+)	(-)	(+)	(+)	(-)	No	n	18.23	2
GP034	7.2	57	IDC	1	2	8(35)	(-)	(-)	(+)	(-)	(-)	No	n	36.20	2
GP035	6.5	50	IDC	3.5	2	NA	(+)	(-)	(+)	(+)	(-)	No	NA	NA	1
GP036	7.3	70	IDC	3	2	42(44)	(+)	(-)	(+)	(-)	(-)	No	n	72.97	2
GP037	5.8	61	IDC	2.4	2	2(18)	(-)	(-)	(+)	(-)	(+)	No	y	41.37	2
GP038	7.8	63	IDC	2.3	3	0(18)	(-)	(-)	(+)	(-)	(-)	No	n	61.10	1
GP039	7.6	59	IDC	4	3	1(22)	(-)	(-)	(+)	(-)	(-)	No	y	26.47	2
GP040	6.0	65	IDC	2.7	3	4(17)	(+)	(-)	(+)	(-)	(+)	No	n	73.03	2
GP041	7.6	43	IDC	1.5	3	4(13)	(+)	(-)	(+)	(+)	(-)	No	y	54.17	1
GP042	7.0	69	IDC	2.5	2	7(13)	(-)	(-)	(+)	(-)	(-)	No	n	70.27	1
GP043	7.5	42	IDC	2.9	3	2(27)	(+)	(-)	(+)	(+)	(-)	No	n	73.07	1
GP044	6.6	57	IDC	4.7	3	7(15)	(+)	(+)	(-)	(-)	(+)	No	n	51.00	3
GP045	7.5	46	IDC	2.2	3	2(17)	(+)	(+)	(-)	(-)	(+)	No	n	61.23	3
GP046	8.4	65	IDC	1.5	2	1(2)	(+)	(-)	(+)	(+)	(-)	No	n	57.37	1
GP047	8.9	35	IDC	6	2	1(18)	(+)	(+)	(+)	(-)	(-)	No	n	58.83	2

GP048	8.2	73	IDC	6	1	0(9)	(-)	(-)	(+)	(+)	(-)	No	n	57.37	1
GP049	7.9	44	IDC	2.65	3	0(3)	(-)	(-)	(-)	(-)	(-)	Yes	n	50.67	3
GP050	7.0	57	IDC	1.3	3	2(14)	(+)	(-)	(+)	(-)	(-)	No	n	66.27	2
GP051	7.3	71	IDC	5	2	1(11)	(+)	(-)	(+)	(+)	(-)	No	n	31.83	1
GP052	6.9	54	IDC	3.9	3	1(17)	(+)	(-)	(-)	(-)	(+)	No	y	41.87	3
GP053	6.6	47	IDC/Lobular	6	2	1(2)	(-)	(-)	(+)	(+)	(-)	No	n	42.10	1
GP054	7.9	54	IDC	2.5	2	1(22)	(+)	(-)	(+)	(+)	(-)	No	n	26.73	2
GP055	9.4	69	IDC	2.9	2	1(16)	(+)	(-)	(+)	(+)	(-)	No	n	36.03	1
GP056	7.5	45	IDC	1.7	3	0(2)	(+)	(-)	(-)	(-)	(+)	No	n	35.47	3
GP057	7	49	ILC	15	2	5(15)	(-)	(-)	(+)	(+)	(-)	No	n	36.67	1
GP058	8.3	59	IDC	1.6	1	1(17)	(+)	(-)	(+)	(+)	(-)	No	n	34.83	1
GP059	8.3	76	IDC	2.5	2	0(1)	(+)	(-)	(+)	(-)	(-)	No	n	39.90	2
GP060	7	53	IDC	2.2	3	0(6)	(-)	(-)	(-)	(-)	(-)	Yes	n	41.03	3
GP061	7.4	46	IDC	2.4	2	0(4)	(-)	(+)	(+)	(+)	(-)	No	n	36.50	1
GP062	7.1	73	IDC	1.7	2	0(2)	(-)	(-)	(+)	(+)	(-)	No	n	36.97	1
GP063	7.5	67	IDC	4	3	3(30)	(-)	(-)	(+)	(-)	(-)	No	n	22.63	2
GP064	6.8	45	IDC	0.9	2	0(5)	(+)	(+)	(+)	(+)	(-)	No	n	35.03	1
GP065	6.9	62	IDC	1.9	3	0(1)	(-)	(-)	(-)	(-)	(-)	Yes	n	38.30	3
GP066	8.1	73	IDC	1.5	1	1(5)	(-)	(-)	(+)	(+)	(-)	No	n	37.67	1
GP067	8.8	51	IDC	2.2	3	1(17)	(-)	(+)	(+)	(+)	(-)	No	n	37.90	2
GP068	6.5	72	IDC	1.5	2	1(13)	(-)	(-)	(+)	(+)	(-)	No	n	32.13	1
GP069	7.5	58	ILC	8.8	2	5(49)	(-)	(-)	(+)	(+)	(-)	No	n	33.40	2
GP070	9.2	41	IDC	1.4	2	1(14)	(-)	(-)	(+)	(-)	(-)	No	n	28.40	2
GP071	7.1	55	ILC	16.1	2	0(23)	(-)	(-)	(+)	(-)	(-)	No	n	28.50	1
GP072	8.5	40	IDC	2	2	3(17)	(+)	(+)	(+)	(+)	(-)	No	n	26.37	2
GP073	8.8	60	IDC	1.3	2	1(23)	(-)	(-)	(+)	(+)	(-)	No	n	24.67	2
GP074	9	32	IDC	2.6	3	1(13)	(+)	(-)	(+)	(-)	(-)	No	n	37.00	2
GP075	8.4	65	IDC	1.8	2	1(17)	(+)	(-)	(+)	(+)	(-)	No	n	37.20	1
GP076	8.8	46	ILC	2.3	2	1(21)	(-)	(-)	(+)	(+)	(-)	No	n	32.73	1
GP077	8.8	52	IDC	2	3	0(2)	(-)	(+)	(+)	(-)	(-)	No	n	36.07	2
GP078	7.9	58	IDC	3	3	2(18)	(-)	(-)	(+)	(+)	(-)	No	n	1.80	3
GP079	7.4	58	IDC	0.8	1	0(1)	(-)	(-)	(+)	(+)	(-)	No	n	26.00	1
GP080	8.7	58	IDC	0.3	2	1(5)	(-)	(-)	(+)	(-)	(-)	No	n	25.70	2
GP082	7.3	36	IDC	3.4	3	0(3)	(-)	(-)	(-)	(-)	(-)	Yes	n	32.40	3
GP083	8.6	76	IDC	2.7	3	2(18)	(+)	(-)	(+)	(+)	(-)	No	n	36.20	2
GP084	8.5	51	IDC	2.7	3	1(11)	(-)	(-)	(+)	(+)	(-)	No	n	1.43	2
GP085	9.4	47	IDC	2.8	3	1(2)	(-)	(+)	(+)	(+)	(-)	No	n	14.90	2
GP086	9.2	60	IDC	1.5	2	0(2)	(+)	(+)	(+)	(-)	(-)	No	n	23.97	2
GP087	9.2	68	IDC	2.4	3	0(3)	(-)	(-)	(-)	(-)	(+)	No	n	24.67	3
GP088	9.2	59	IDC	2.7	3	0(1)	(-)	(-)	(-)	(-)	(+)	No	n	18.63	3
GP089	6.3	71	IDC	2.4	2	0(5)	(-)	(+)	(-)	(-)	(+)	No	n	33.33	3
GP094	7.2	57	IDC	1.5	1	0(3)	(-)	(-)	(+)	(-)	(-)	No	n	37.37	1
GP096	8.6	53	ILC	0.8	2	0(5)	(+)	(-)	(+)	(+)	(-)	No	n	35.63	2
GP097	9.3	35	IDC	5.9	3	6(19)	(+)	(+)	(+)	(+)	(-)	No	n	15.97	2
GP098	6.9	59	IDC	1	3	0(2)	(-)	(+)	(+)	(-)	(+)	No	n	36.10	1
GP099	8.8	47	IDC	1.9	2	1(19)	(-)	(-)	(+)	(+)	(-)	No	n	35.60	2
GP100	9.0	68	IDC	1.4	2	0(3)	(-)	(-)	(-)	(-)	(-)	Yes	n	33.20	3
GP101	9.5	35	IDC	2.6	2	2(5)	(+)	(-)	(-)	(-)	(+)	No	n	32.60	3
GP102	9.2	55	IDC	2.9	3	0(3)	(+)	(-)	(-)	(-)	(+)	No	n	15.80	3
GP103	9.0	75	IDC	2.3	3	1(4)	(-)	(-)	(-)	(-)	(+)	No	n	34.57	3
GP104	7.4	47	IDC	2.5	3	3(24)	(-)	(-)	(+)	(+)	(-)	No	y	33.53	2
GP105	9.3	64	IDC	3	3	2(38)	(+)	(+)	(+)	(+)	(-)	No	n	25.47	2
GP106	8.1	66	IDC	2.3	2	1(19)	(+)	(-)	(+)	(+)	(+)	No	n	28.93	1

GP107	6.5	63	IDC	1.6	3	0(5)	(-)	(-)	(-)	(-)	(-)	Yes	n	30.73	3
GP109	6.7	53	IDC	3.5	3	2(19)	(-)	(+)	(-)	(-)	(+)	No	n	33.70	3
GP110	9.6	61	IDC	2.2	3	0(2)	(-)	(-)	(+)	(+)	(-)	No	n	30.33	2
GP111	7.3	69	IDC	1.3	2	3(10)	(-)	(-)	(+)	(+)	(-)	No	n	33.17	2
GP112	5.6	66	ILC	2.1	2	2(18)	(-)	(-)	(+)	(+)	(-)	No	n	28.50	1
GP113	7.4	50	IDC/ILC	2.6	3	0(3)	(+)	(-)	(-)	(-)	(+)	No	n	27.10	3
GP114	9.1	62	IDC	2.5	3	2(12)	(+)	(-)	(+)	(+)	(-)	No	n	27.97	2
GP115	9.0	45	IDC	2.2	3	2(17)	(+)	(+)	(+)	(+)	(+)	No	n	34.03	2
GP116	6.4	85	IDC	1.5	2	0(2)	(-)	(-)	(+)	(+)	(-)	No	n	20.40	1
GP117	8.3	38	IDC	4.5	3	2(10)	(-)	(+)	(-)	(-)	(+)	No	n	27.23	3
GP119	5.8	77	ILC	2.4	2	0(2)	(-)	(-)	(+)	(+)	(-)	No	n	26.20	1
GP121	7.2	53	IDC	2.6	3	1(15)	(+)	(-)	(+)	(-)	(-)	No	n	23.20	2
GP122	6.1	34	IDC	2.5	2	0(3)	(-)	(-)	(+)	(-)	(-)	No	n	20.10	2
GP123	7.3	67	IDC	2.5	3	0(2)	(-)	(-)	(+)	(+)	(-)	No	n	27.33	2
GP124	7.9	41	IDC	1.1	3	0(2)	(-)	(-)	(-)	(-)	(-)	Yes	n	29.93	3
GP125	8.2	60	IDC	3	3	0(2)	(-)	(-)	(-)	(-)	(+)	No	y	17.93	3
GP127	7.7	59	IDC	2.8	3	0(4)	(-)	(-)	(+)	(+)	(-)	No	n	21.67	2
GP128	7.8	65	IDC	2.4	3	0(4)	(-)	(-)	(+)	(-)	(-)	No	n	29.47	2
GP129	8.7	73	IDC	2	2	0(1)	(-)	(-)	(+)	(+)	(-)	No	n	26.53	2
GP130	6.7	50	IDC	1.1	1	0(2)	(-)	(+)	(+)	(-)	(-)	No	n	31.57	1
GP131	8.5	46	IDC	1.8	3	5(35)	(+)	(-)	(-)	(-)	(-)	Yes	n	29.47	3
GP132	9.4	65	IDC	2.5	3	2(14)	(+)	(-)	(+)	(+)	(-)	No	n	11.00	2
GP133	6.7	59	IDC	10.8	3	0(0)	(+)	(-)	(+)	(-)	(-)	No	n	32.00	2
GP134	6.8	55	IDC	3	3	0(6)	(-)	(-)	(-)	(-)	(-)	Yes	n	26.40	3
GP135	5.7	61	IDC	2	2	16(24)	(-)	(-)	(+)	(+)	(-)	No	n	27.57	2
GP136	8.3	48	IMC	3.2	2	0(7)	(-)	(+)	(+)	(+)	(-)	No	n	32.00	2
GP137	6.9	48	IDC	6	3	12(20)	(+)	(-)	(-)	(-)	(+)	No	y	28.87	3
GP138	7.2	49	IDC	1.6	2	1(20)	(-)	(-)	(+)	(+)	(-)	No	n	22.70	2
GP139	7.8	75	ILC	7	3	3(17)	(+)	(-)	(+)	(+)	(-)	No	n	29.60	2
GP140	8.8	42	IDC	2.4	2	1(15)	(-)	(-)	(+)	(-)	(-)	No	n	18.37	2
GP141	8.0	52	IDC	3	3	1(3)	(+)	(-)	(+)	(-)	(+)	No	n	25.87	2
GP142	7.3	54	IDC	2.1	3	0(3)	(-)	(-)	(+)	(+)	(-)	No	n	25.07	2
GP143	8.4	53	IDC	3.4	3	0(3)	(+)	(-)	(-)	(-)	(+)	No	y	16.00	3
GP144	7.5	53	IDC	3.6	3	14(21)	(-)	(-)	(-)	(-)	(+)	No	n	28.97	3
GP145	7.2	48	IMC	7.5	2	14(19)	(-)	(-)	(+)	(+)	(-)	No	n	29.63	2
GP146	6.2	48	IDC	1.7	2	5(14)	(+)	(-)	(+)	(+)	(-)	No	n	29.23	1
GP147	7.3	57	IDC	1.2	3	0(2)	(-)	(-)	(+)	(+)	(-)	No	n	29.47	2
GP148	7.9	51	IDC	4	3	2(21)	(+)	(-)	(+)	(+)	(-)	No	n	22.83	2
GP149	8.6	30	IDC	2.4	3	2(18)	(+)	(-)	(+)	(-)	(-)	No	n	30.57	2
GP150	8.0	60	IDC	1.6	1	0(1)	(-)	NA	(+)	(-)	(-)	No	n	28.83	2
GP151	7.1	67	IDC	1.2	2	0(5)	(+)	(-)	(+)	(-)	(-)	No	n	18.77	1
GP152	7.5	72	IDC	2.1	2	0(3)	(+)	(-)	(+)	(+)	(-)	No	n	30.13	2
GP153	7.8	43	IDC	2.3	2	0(2)	(-)	(-)	(-)	(-)	(-)	Yes	n	26.30	3
GP154	8.3	66	IDC	1.9	3	0(4)	(-)	(-)	(+)	(-)	(-)	No	n	27.33	2
GP155	5.6	69	IDC	1.8	3	0(1)	(-)	NA	(-)	(-)	(-)	Yes	n	26.53	3
GP156	8.7	52	IDC	2.1	1	0(2)	(-)	(-)	(+)	(+)	(-)	No	n	21.47	1
GP157	7.9	45	IDC	3.2	2	4(20)	(+)	(-)	(+)	(-)	(-)	No	n	26.13	2
GP158	7.8	78	IDC	1.4	3	0(1)	(-)	NA	(-)	(-)	(-)	Yes	n	25.53	3
GP159	7.3	58	IDC	1.4	2	0(3)	(-)	(-)	(+)	(+)	(-)	No	n	27.03	1
GP160	8.3	81	IDC	1.5	2	2(17)	(+)	(-)	(+)	(+)	(-)	No	n	25.47	2
GP161	8.3	73	IDC	0.8	2	0(1)	(+)	(+)	(+)	(+)	(-)	No	n	22.03	2

The estrogen receptor (ER), progesterone receptor (PR) and Her2/neu (Her2) status were evaluated by immunohistochemistry or by fluorescence in situ hybridization using standard clinical protocols.
