# Supplementary material for: A new gene expression signature, the ClinicoMolecular Triad Classification, may improve prediction and prognostication of breast cancer at the time of diagnosis
Source: Breast Cancer Res. 2011 Sep 22;13(5):R92. doi: 10.1186/bcr3017 (PMC3262204; doi:10.1186/bcr3017)
Supplement: Additional file 2 — Supplementary Table S2 Summary of resource, platform, adjuvant treatment status and clinical end point of the microarray data sets used in this study. DMFS = distant metastasis-free survival; RFS = relapse-free survival. [file bcr3017-S2.PDF]

Table S2 Microarray dataset resource

Data cohorts	GEO accessions* or other availability	Tumor No. in the dataset	Used No. in the study †	Contained adjuvant treatment	Clinical endpoint	Microarray platform	Reference
**Training cohort**							
	GSE16987	161	149	No	RFS	Illumina HumanRef-8 V2	This study
**Validation cohort**							
	See URL links#	295	295	Yes	DMFS	Agilent Hu25K	1,2
	GSE1456	159	159	Yes	RFS	Affymetrix U133 A and B	3
	GSE2034	286	286	No	RFS	Affymetrix U133 A	4
	GSE2990 GSE6532	414	380	Yes	RFS	Affymetrix U133 A and B	5,6,7
	GSE3494 GSE4922	251	240	Yes	RFS	Affymetrix U133 A and B	8,9
	GSE7390	198	119	No	RFS	Affymetrix U133 A	10
	GSE9195	77	77	Yes	RFS	Affymetrix U133 Plus2	11
	GSE10886 GSE6128	245	245	Yes	RFS	Agilent H1A UNC custom (GPL1390)	12,13
	GSE11121	200	186	No	DMFS	Affymetrix U133 A	14
	GSE20194 (GSE16716)	278	248	Yes	pCR	Affymetrix U133 A	15
	GSE21653	266	252	Yes	DFS	Affymetrix U133 Plus2	16

*GEO data are available at: http://www.ncbi.nlm.nih.gov/projects/geo/

†Only individual cases with followed-up data in the validation cohort were included.

# http://www.rii.com/publications/2002/nejm.html and http://microarray-pubs.stanford.edu/wound_NKI/
