# Supplementary material for: A new gene expression signature, the ClinicoMolecular Triad Classification, may improve prediction and prognostication of breast cancer at the time of diagnosis
Source: Breast Cancer Res. 2011 Sep 22;13(5):R92. doi: 10.1186/bcr3017 (PMC3262204; doi:10.1186/bcr3017)
Supplement: Additional file 3 — Supplementary methods. File containing descriptions of microarray data resources, platform-specific data treatment and analyses, integration of published gene expression signatures and signaling pathway signatures, and generation of gene expression profiles for the Her2+/TN phenotype. ANOVA = analysis of variance; ER = estrogen receptor; ESR1 = estrogen receptor 1 gene; GEO = Gene Expression Omnibus; Her2 = human epidermal growth factor receptor 2; MAQC = MicroArray Quality Control project; PAM50 = 50-gene prediction analysis of microarray; PM-MM = Pairs of Perfect Match(PM) and Mismatch(MM) oligonucleotide probes; PR = progesterone receptor; TN = triple-negative; WS = wound-response gene signature. [file bcr3017-S3.PDF]

Supplemental methods

Microarray data resources. The primary dataset generated by using Illumina HumanRef-8 v2 Expression BeadChip (http://www.illumina.com/). Total 161 breast tumors were taken between 2006 and 2008 from Princess Margaret Hospital and Mount Sinai Hospital (Toronto, ON) and finally, 149 invasive breast cancers were created as the training cohort (Table S1). The information for the validation microarray datasets [1-16] was listed in Table S2. The microarray data and their patient clinical information for the validation dataset with 295 breast cancers from Netherlands Cancer Institute [1,2] were downloaded from websites http://www.rii.com/publications/2002/nejm.html and http://microarray-pubs.stanford.edu/wound_NKI/. The other validation datasets were downloaded from NCBI Gene Expression Omnibus website http://www.ncbi.nlm.nih.gov/geo, using the accession numbers from the respective studies. All microarray data used in this study excluded replicated cases and contained clinical endpoint information. Any type of recurrence, including local recurrence and distant metastasis, was used to analyzed the relapse-free survival. All tumors must come with their clinical ER, PR and Her2 status. If the status is not available from the published materials, a request would be sent to the author, or array expression values of the three genes were used.

Agilent microarray data processing. The downloaded Agilent Hu25K data for the 295 breast cancers came with log ratios of the signals for each probe from the tumor relative to pooled sample from all patients [1,2]. The downloaded GEO series matrix files from two Agilent datasets of GSE10886 [12] and GSE6128 [13] were in $\log_2$ ratios of the tumor RNA relative to a modified Stratagene Human Universal Reference RNA, and only arrays in platform GPL1390 were used in the study. To make the two Agilent

datasets compatible with other microarray datasets, the log ratios of Agilent Hu25K dataset were converted to log_2 ratios; whereas the log_2 ratios of GSE10886 and GSE6128 datasets was first converted back to ratios and then compared that to the average ratios of all the probes in log_2 format.

Affymetrix microarrays data processing. The downloaded Affymetrix CEL data were processed by Expression Console version 1.1.1 of the GeneChip Operating Software (Affymetrix Inc., Santa Clara, CA). The Probe Logarithmic Intensity Error Estimation method was used to produce a summary value for each probe set by Quantile normalization and PM-MM protocols. The downloaded GEO series matrix files in normalized intensity values were directly used in next step of data processing. A value of 16 was assigned to any normalized intensity value that was less than 16, according to the recommendation from MAQC Consortium [17]. A log_2 expression ratio of an intensity value to the average signal value for each transcript in all samples was calculated.

Integration of published gene expression signatures. Sixteen gene expression signatures that have previously been reported to have prognostic predictability in breast cancers [1,4,5,8,12,14,19-27] are summarized in Table S3. Out of the 16 gene signatures, 14 microarray-based signatures were used to compare and evaluate the gene signature generated in this study. All array probes in the 14 signatures were re-annotated by using the tools in http://www.ncbi.nlm.nih.gov, then their official gene symbols were used to search each array data from every tumor in the training and validation cohorts. All probes that matched to a specific gene symbol were used to classify the tumors. The expression centroid values for each gene in the signatures were used to score the validating data series. The centroid data for PAM50 [12] was

available at https://genome.unc.edu/pubsup/breastGEO. If a centroid data was not available in their published materials, -1 was used as the good signature centroid value and +1 for poor signature. A Pearson correlation was calculated to get the quantitative scores of corresponding expression values for the genes in each tumor to the expression centroid values of the genes in each prognostic signature. The classification of Subtype [25], PAM50 [12] and CMTC, the gene signature generated in this study, were based on the nearest expression centroid method [2,25]. The adjusted threshold value of correlation coefficient -0.15 was used for WS [2,27], and 0.4 for 70GS [1,2]. The correlation coefficient value of zero was used as threshold value to classify the validation tumors for other signatures.

Integration of published signaling pathway signatures. Nineteen pathway signatures that enable integration of patterns in predicting activity for oncogenic signaling and other cellular pathways were collected. The training data and methods to develop gene expression signatures for pathway activity have been previously described [28,29]. To test the probabilities of the pathway activity in the 149 breast cancers in training cohort, the predicted activity patterns for the 19 pathways were represented into the three types in CMTC that was generated in this study by using a hierarchical clustering. A Pearson correlation was performed to depict the co-regulation among the pathways.

Statistics and data analysis. All microarray data were represented as $\log_2$ ratios for the expression analysis of gene transcription and entered into the Acuity software version 4 (Molecular Devices, Sunnyvale, CA) with their annotation files and clinical information for data analysis. Variant significance *t* test and ANOVA test were used to evaluate the differential expression between cancer groups. A Benjamini-Hochberg

method was used to control false discovery rate, and the most conservative correction method Bonferroni was applied to the *P* values of corresponding *t* tests between different microarray expression patterns. Chi-square test and Fisher's exact test were used to test the significance of the clinical and pathological variables between different cancer types. The hierarchical analysis was used to generate and present the expression patterns. Kaplan-Meier analysis was used to compare patient's survivals in differential gene expression groups, and their differences were determined by the Log-rank Test. Univariate and multivariate analyses of prognostic factors were performed by using Cox proportional hazard method. Receiver Operating Characteristic analysis was used to score the Area Under the Curve. All reported *P* values were two-sided, and a *P* value of less than 0.05 was considered statistically significant.

Illumina array quality measures and data processing. To measure the quality of the Illumina microarray, we incorporated a control RNA sample using Universal Human Reference RNA (Stratagene; La Jolla, CA) into each of the 30 Illumina BeadChips. The Reference microarray dataset is available at GEO website with the accession number GSE16984. For each of the 22,184 unique probes in the dataset, there was an average of 42.3±8.1 replicated beads. The correlation analysis of the expression intensity values revealed a very high average correlation coefficient of 0.9908±0077 among the 30 controls. In the sample specimens, the average correlation coefficient was 0.9918±0108 for the 10 pairs of duplicated fine needle aspiration biopsies taken from the same tumors and 0.8491±0407 among different tumors. All duplicates of the cancer samples were combined for each tumor, and a total 149 microarray data of breast cancers was used for next analysis for the selected 149 invasive breast cancers. By adjusting the lowest intensity value, 713 probes with a $\log_2$ ratio value of "0" across all samples were considered as under detectable signals and were eliminated from the next step of the

analysis. Respectively, within the 149 breast cancers, the expression levels of ESR1 and ERBB2 from microarray were consistent very well with clinical ER and Her2 status measured by immunohistochemistry or fluorescent in situ hybridization ($P < 0.0001$).

Generation of gene expression profile for Her2+/TN phenotype. Of the 149 breast cancers in the training cohort, 44 were Her2-positive (Her2+) or triple negative (TN, ER-/PR-/Her2-). The 44 Her2+/TN tumors were used as a group to distinguish the gene expression pattern compared to the other 105 tumors. A *t* test was performed to screen the most differentially expressed genes between the two groups. A total of 1428 probes (representing 1376 genes, some genes were represented by multiple oligonucleotide probes in the microarray) were selected at a level of Bonferroni corrected *P* value less than 0.01. The hierarchical clustering analysis using the 1428-probe set resulted in division of a group of 39 tumors with 36 Her2+/TN status from the other group of 110 tumors with 8 Her2+/TN status . As shown in Figure S1A, the group with less Her2+/TN tumors can visibly be separated into two subgroups which we labeled as group 1 and 2 according to the gene expression profile, and the group enriched with Her2+/TN tumors was shown as group 3. Because we wanted to look for the molecular basis of dividing breast cancers into 3 groups similar to oncologists in the clinical settings, we went on to perform a second screen using all the differentially expressed genes that were best in separating the 149 breast cancers of the training cohort into three clusters with most Her2+/TN in one group. A total of1349 probes (1304 genes) were selected at a level of the *P* value less than 0.001 by an ANOVA test among the three groups. As a result, a more apparent three-cluster pattern was seen using the 1349-probe set (Figure S1B). Out of the 42 tumors in group 1, only one wasHer2+/TN; there are 7 Her2+/TN in the 68 tumors of group 2, and 36 Her2+/TN in the 39 tumors of group 3.

References

1. van de Vijver MJ, He YD, van't Veer LJ, Dai H, Hart AA, Voskuil DW, Schreiber GJ, Peterse JL, Roberts C, Marton MJ, Parrish M, Atsma D, Witteveen A, Glas A, Delahaye L, van der Velde T, Bartelink H, Rodenhuis S, Rutgers ET, Friend SH, Bernards R: **A gene-expression signature as a predictor of survival in breast cancer.** *N Engl J Med* 2002, **347**:1999-2009.
2. Chang HY, Nuyten DS, Sneddon JB, Hastie T, Tibshirani R, Sørlie T, Dai H, He YD, van't Veer LJ, Bartelink H, van de Rijn M, Brown PO, van de Vijver MJ: **Robustness, scalability, and integration of a wound-response gene expression signature in predicting breast cancer survival.** *Proc Natl Acad Sci USA* 2005, **102**:3738-3743.
3. Pawitan Y, Bjöhle J, Amler L, Borg AL, Egyhazi S, Hall P, Han X, Holmberg L, Huang F, Klaar S, Liu ET, Miller L, Nordgren H, Ploner A, Sandelin K, Shaw PM, Smeds J, Skoog L, Wedrén S, Bergh J: **Gene expression profiling spares early breast cancer patients from adjuvant therapy: derived and validated in two population-based cohorts.** *Breast Cancer Res* 2005, **7**:R953-964.
4. Wang Y, Klijn JG, Zhang Y, Sieuwerts AM, Look MP, Yang F, Talantov D, Timmermans M, Meijer-van Gelder ME, Yu J, Jatkoe T, Berns EM, Atkins D, Foekens JA: **Gene-expression profiles to predict distant metastasis of lymph-node-negative primary breast cancer.** *Lancet* 2005, **365**:671-679.
5. Sotiriou C, Wirapati P, Loi S, Harris A, Fox S, Smeds J, Nordgren H, Farmer P, Praz V, Haibe-Kains B, Desmedt C, Larsimont D, Cardoso F, Peterse H, Nuyten D, Buyse M, Van de Vijver MJ, Bergh J, Piccart M, Delorenzi M: **Gene expression profiling in breast cancer: understanding the molecular basis of histologic grade to improve prognosis.** *J Natl Cancer Inst* 2006, **98**:262-272.
6. Loi S, Haibe-Kains B, Desmedt C, Lallemand F, Tutt AM, Gillet C, Ellis P, Harris A, Bergh J, Foekens JA, Klijn JG, Larsimont D, Buyse M, Bontempi G, Delorenzi M, Piccart MJ, Sotiriou C: **Definition of clinically distinct molecular subtypes in estrogen receptor-positive breast carcinomas through genomic grade.** *J Clin Oncol* 2007, **25**:1239-1246.
7. Loi S, Haibe-Kains B, Desmedt C, Wirapati P, Lallemand F, Tutt AM, Gillet C, Ellis P, Ryder K, Reid JF, Daidone MG, Pierotti MA, Berns EM, Jansen MP, Foekens JA, Delorenzi M, Bontempi G, Piccart MJ, Sotiriou C: **Predicting prognosis using molecular profiling in estrogen receptor-positive breast cancer treated with tamoxifen.** *BMC Genomics* 2008, **9**:239.
8. Miller LD, Smeds J, George J, Vega VB, Vergara L, Ploner A, Pawitan Y, Hall P, Klaar S, Liu ET, Bergh J: **An expression signature for p53 status in human breast cancer predicts mutation status, transcriptional effects, and patient survival.** *Proc Natl Acad Sci USA* 2005, **102**:13550-13555.
9. Ivshina AV, George J, Senko O, Mow B, Putti TC, Smeds J, Lindahl T, Pawitan Y, Hall P, Nordgren H, Wong JE, Liu ET, Bergh J, Kuznetsov VA, Miller LD: **Genetic reclassification of histologic grade delineates new clinical subtypes of breast cancer.** *Cancer Res* 2006, **66**:10292-10301.

10. Desmedt C, Piette F, Loi S, Wang Y, Lallemand F, Haibe-Kains B, Viale G, Delorenzi M, Zhang Y, d'Assignies MS, Bergh J, Lidereau R, Ellis P, Harris AL, Klijn JG, Foekens JA, Cardoso F, Piccart MJ, Buyse M, Sotiriou C; TRANSBIG Consortium: **Strong time dependence of the 76-gene prognostic signature for node-negative breast cancer patients in the TRANSBIG multicenter independent validation series.** *Clin Cancer Res* 2007, **13**:3207-3214.

11. Loi S, Haibe-Kains B, Majjaj S, Lallemand F, Durbecq V, Larsimont D, Gonzalez-Angulo AM, Pusztai L, Symmans WF, Bardelli A, Ellis P, Tutt AN, Gillett CE, Hennessy BT, Mills GB, Phillips WA, Piccart MJ, Speed TP, McArthur GA, Sotiriou C: **PIK3CA mutations associated with gene signature of low mTORC1 signaling and better outcomes in estrogen receptor-positive breast cancer.** *Proc Natl Acad Sci USA* 2010, **107**:10208-10213.

12. Parker JS, Mullins M, Cheang MC, Leung S, Voduc D, Vickery T, Davies S, Fauron C, He X, Hu Z, Quackenbush JF, Stijleman IJ, Palazzo J, Marron JS, Nobel AB, Mardis E, Nielsen TO, Ellis MJ, Perou CM, Bernard PS: **Supervised risk predictor of breast cancer based on intrinsic subtypes.** *J Clin Oncol* 2009, **27**:1160-1167.

13. Hoadley KA, Weigman VJ, Fan C, Sawyer LR, He X, Troester MA, Sartor CI, Rieger-House T, Bernard PS, Carey LA, Perou CM: **EGFR associated expression profiles vary with breast tumor subtype.** *BMC Genomics* 2007, **8**:258.

14. Schmidt M, Böhm D, von Törne C, Steiner E, Puhl A, Pilch H, Lehr HA, Hengstler JG, Kölbl H, Gehrmann M: **The humoral immune system has a key prognostic impact in node-negative breast cancer.** *Cancer Res* 2008, **68**:5405-5413.

15. Juul N, Szallasi Z, Eklund AC, Li Q, Burrell RA, Gerlinger M, Valero V, Andreopoulou E, Esteva FJ, Symmans WF, Desmedt C, Haibe-Kains B, Sotiriou C, Pusztai L, Swanton C: **Assessment of an RNA interference screen-derived mitotic and ceramide pathway metagene as a predictor of response to neoadjuvant paclitaxel for primary Triple-negative breast cancer: a retrospective analysis of five clinical trials.** *Lancet Oncol* 2010, **11**:358-365.

16. Sabatier R, Finetti P, Cervera N, Lambaudie E, Esterni B, Mamessier E, Tallet A, Chabannon C, Extra JM, Jacquemier J, Viens P, Birnbaum D, Bertucci F: **A gene expression signature identifies two prognostic subgroups of basal breast cancer.** *Breast Cancer Res Treat* 2011, **126**:407-420.

17. MAQC Consortium: **The MicroArray Quality Control (MAQC) project shows inter- and intraplatform reproducibility of gene expression measurements.** *Nat Biotechnol* 2006, **24**:1151-1161.

18. Loberg RD, Bradley DA, Tomlins SA, Chinnaiyan AM, Pienta KJ: **The lethal phenotype of cancer: the molecular basis of death due to malignancy.** *CA Cancer J Clin* 2007, **57**:225-241.

19. Shipitsin M, Campbell LL, Argani P, Weremowicz S, Bloushtain-Qimron N, Yao J, Nikolskaya T, Serebryiskaya T, Beroukhim R, Hu M, Halushka MK, Sukumar S, Parker LM, Anderson KS, Harris LN, Garber JE, Richardson AL, Schnitt SJ, Nikolsky Y, Gelman RS, Polyak K: **Molecular definition of breast tumor heterogeneity.** *Cancer Cell* 2007, **11**:259-273.

20. Oh DS, Troester MA, Usary J, Hu Z, He X, Fan C, Wu J, Carey LA, Perou CM: **Estrogen-regulated genes predict survival in hormone receptor-positive breast cancers.** *J Clin Oncol* 2006, **24**:1656-1664.

21. Ben-Porath I, Thomson MW, Carey VJ, Ge R, Bell GW, Regev A, Weinberg RA: **An embryonic stem cell-like gene expression signature in poorly differentiated aggressive human tumors.** *Nat Genet* 2008, **40**:499-507.

22. Liu R, Wang X, Chen GY, Dalerba P, Gurney A, Hoey T, Sherlock G, Lewicki J, Shedden K, Clarke MF: **The prognostic role of a gene signature from tumorigenic breast-cancer cells.** *N Engl J Med* 2007, **356**:217-226.

23. Paik S, Shak S, Tang G, Kim C, Baker J, Cronin M, Baehner FL, Walker MG, Watson D, Park T, Hiller W, Fisher ER, Wickerham DL, Bryant J, Wolmark N: **A multigene assay to predict recurrence of tamoxifen-treated, node-negative breast cancer.** *N Engl J Med* 2004, **351**:2817-2826.

24. Finak G, Bertos N, Pepin F, Sadekova S, Souleimanova M, Zhao H, Chen H, Omeroglu G, Meterissian S, Omeroglu A, Hallett M, Park M: **Stromal gene expression predicts clinical outcome in breast cancer.** *Nat Med* 2008, **14**:518-527.

25. Sorlie T, Tibshirani R, Parker J, Hastie T, Marron JS, Nobel A, Deng S, Johnsen H, Pesich R, Geisler S, Demeter J, Perou CM, Lønning PE, Brown PO, Børresen-Dale AL, Botstein D: **Repeated observation of breast tumor subtypes in independent gene expression data sets.** *Proc Natl Acad Sci USA* 2003, **100**:8418-8423.

26. Bierie B, Chung CH, Parker JS, Stover DG, Cheng N, Chytil A, Aakre M, Shyr Y, Moses HL: **Abrogation of TGF-beta signaling enhances chemokine production and correlates with prognosis in human breast cancer.** *J Clin Invest* 2009, **119**:1571-1582.

27. Chang HY, Sneddon JB, Alizadeh AA, Sood R, West RB, Montgomery K, Chi JT, van de Rijn M, Botstein D, Brown PO: **Gene expression signature of fibroblast serum response predicts human cancer progression: similarities between tumors and wounds.** *PLoS Biol* 2004, **2**:E7.

28. Bild AH, Yao G, Chang JT, Wang Q, Potti A, Chasse D, Joshi MB, Harpole D, Lancaster JM, Berchuck A, Olson JA Jr, Marks JR, Dressman HK, West M, Nevins JR: **Oncogenic pathway signatures in human cancers as a guide to targeted** therapies. *Nature* 2006, 439:353-357.

29. Gatza ML, Lucas JE, Barry WT, Kim JW, Wang Q, Crawford MD, Datto MB, Kelley M, Mathey-Prevot B, Potti A, Nevins JR: **A pathway-based classification of human breast cancer.** *Proc Natl Acad Sci USA* 2010, **107**:6994-6999.
