# Supplementary material for: A new gene expression signature, the ClinicoMolecular Triad Classification, may improve prediction and prognostication of breast cancer at the time of diagnosis
Source: Breast Cancer Res. 2011 Sep 22;13(5):R92. doi: 10.1186/bcr3017 (PMC3262204; doi:10.1186/bcr3017)
Supplement: Additional file 4 — Supplementary Figure S1 Generation of gene expression profile for Her2+/TN phenotype in the training cohort (n = 149). (A) First screening of Her2+/TN-related genes. A group of 44 Her2+/TN breast cancers were used to distinguish the gene expression from the other 105 tumors. A total of 1,428 probes were selected at a level of the Bonferroni-corrected P value < 0.01. By using the 1,428-probe set in a hierarchical clustering pattern, 39 tumors that were mostly Her2+/TN formed group 3, with two other subgroups emerging on the heat map: groups 1 and 2. (B) Second screening for the most differentially expressed genes between the three groups. By performing an analysis of variance test, 1,349 probes were selected at a level of P < 0.001 among the three groups. A three-cluster pattern is apparent on the heat map, based on hierarchical clustering analysis using the 1,349-probe set. The tumors with Her2+/TN status were 2.4% (1 of 42) in group 1, 10.3% (7 of 68) in group 2 and 92.3% (36 of 39) in group 3. The bottom color bars represent Her2+ (deep pink) and TN (blue). ANOVA = analysis of variance; Her2 = human epidermal growth factor receptor 2; TN = triple-negative. [file bcr3017-S4.PDF]

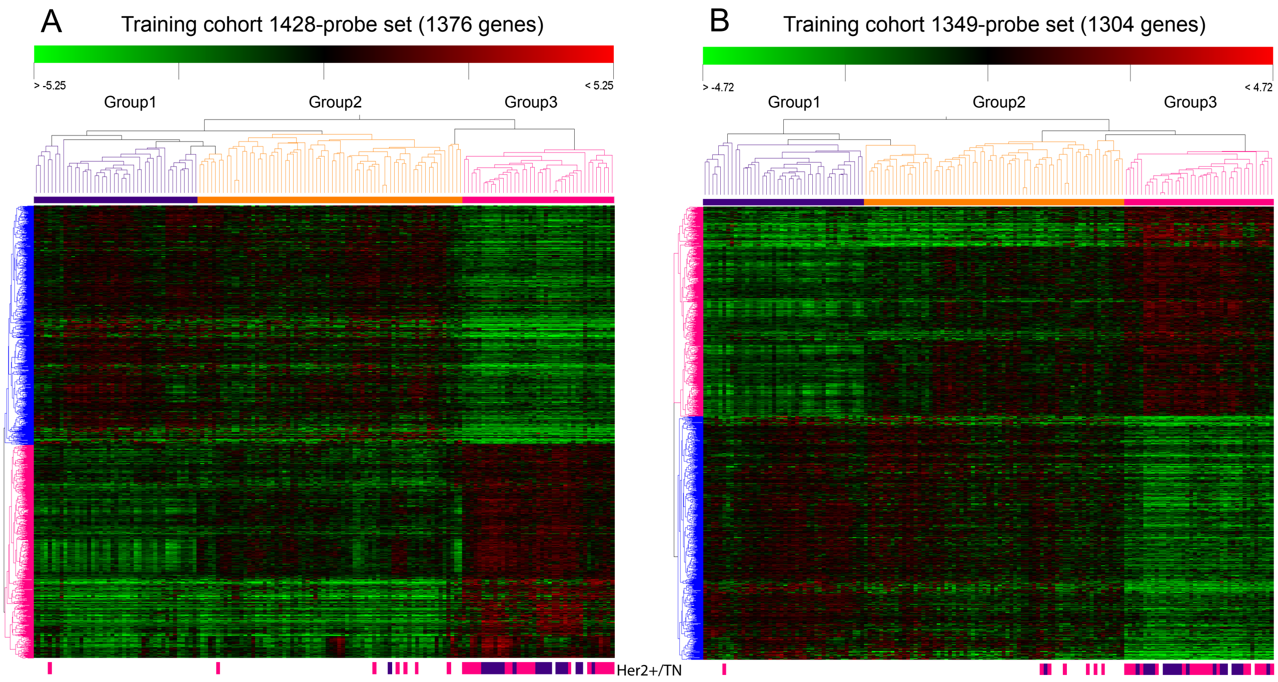

Supplemental figure S1 Generation of gene expression profile for Her2+/TN phenotype in the training cohort (n=149). (**A**). First screening of Her2+/TN related genes. 44 Her2+/TN breast cancers were used as the group to distinguish the gene expression from the other 105 tumors. 1428 probes were selected at a level of the Bonferroni corrected P value less than 0.01. By using the 1428-probe set in a hierarchical clustering pattern, 39 tumors that were mostly Her2+/TN formed group 3 with two other subgroups emerging on the heat map, groups 1 and 2. (**B**) Second screening for the most differentially expressed genes between the three groups. By ANOVA test, 1349 probes were selected at a level of P value less than 0.001 among the three groups. A three-cluster pattern was apparent on the heat map based on hierarchical clustering analysis using the 1349-probe set. The tumors with Her2+/TN status were 2.4% (1/42) in group 1, 10.3% (7/68) in group 2 and 92.3% (36/39) in group 3. The bottom color bars: deep pink, Her2+; blue, TN.
