# Supplementary material for: A new gene expression signature, the ClinicoMolecular Triad Classification, may improve prediction and prognostication of breast cancer at the time of diagnosis
Source: Breast Cancer Res. 2011 Sep 22;13(5):R92. doi: 10.1186/bcr3017 (PMC3262204; doi:10.1186/bcr3017)
Supplement: Additional file 5 — Supplementary Table S3 Summary of name, definition, platform and reference of the prognostic signatures used in this study and the overlapped genes between ClinicoMolecular Triad Classification and published independent breast cancer gene expression prognostic signatures. TGF = transforming growth factor. [file bcr3017-S5.PDF]

Table S3 The ClinicoMolecular Triad Classification (CMTC) and published independent breast cancer gene expression prognostic signatures

Signature name	Signature definition	Platform	Number of probes	Number of known genes	Overlapped gene of in preCMTC	Reference
preCMTC*	Pre-ClinicoMolecular Triad Classification Signature	Illumina	1349	1304	1304	This Study
CMTC*	ClinicoMolecular Triad Classification	Illumina	828	803	803	This Study
37GS	lethal phenotype genes signature	Affymetrix	~	37	11	18
70GS	MammaPrint	Agilent	70	62	26	1,2
76GS	Rotterdam signature	Affymetrix	76	70	10	4
97GS	Genomic Grade Index	Affymetrix	128	108	93	5
CD44	CD44 gene signature	SAGE	~	58	8	19
ERGS	Estrogen-regulated genes expression signature	Agilent	822	769	223	20
ESGS	Embryonic stem cell–like gene signature	Affymetix	1034	1025	106	21
IGS	Invasiveness gene signature	Affymetrix	186	181	29	22
Oncotype	Oncotype DX assay	RT-PCR	~	16	9	23
P53GS	P53 mutation status gene expression signature	Affymetrix	32	23	11	8
PAM50	Prediction analysis of microarray of 50 genes	Agilent	~	50	28	12
Proliferation	Proliferation metagene signature	Affymetrix	97	83	75	14
SDPP	Stroma-derived prognostic predictor	Agilent	163	155	32	24
Subtype	Intrinsic genes subtype	cDNA Array	552	512	92	25
TGFβRII	Type II TGF-β receptor gene signature	Affymetrix	156 (Mouse)	149 (Human)	6	26
WS	Wound-response gene expression signature	cDNA Array	512	462	73	2,27

* The 803 genes in CMTC were derived from the 1304 genes in preCMTC minus 501 overlapped genes from 16 independent prognostic gene signatures.
