# Supplementary material for: A new gene expression signature, the ClinicoMolecular Triad Classification, may improve prediction and prognostication of breast cancer at the time of diagnosis
Source: Breast Cancer Res. 2011 Sep 22;13(5):R92. doi: 10.1186/bcr3017 (PMC3262204; doi:10.1186/bcr3017)
Supplement: Additional file 7 — Supplementary Table S5 Univariate and multivariate analyses of standard clinicopathological parameters, 14 independent gene signatures and CMTC as prognostic indicators for relapse among 1,058 breast cancer patients without adjuvant therapy in the validation cohort. CI = confidence interval; CMTC = ClinicoMolecular Triad Classification; ER = estrogen receptor; ERGS = estrogen-regulated gene expression signature; ESGS = embryonic stem cell-like gene signature; Her2 = human epidermal growth factor receptor 2; IGS = "invasiveness" gene signature; LN = lymph node status; PAM50 = 50-gene prediction analysis of microarray; SDPP = stroma-derived prognostic predictor; TGFβRII = transforming growth factor β receptor type II; TN = triple-negative; WS = wound-response gene signature. [file bcr3017-S7.PDF]

Table S5 Univariate and multivariate analyses of standard clinicopathology parameters, 14 independent gene signatures and CMTC as prognostic indicators for relapse among 1058 breast cancer patients without adjuvant therapy in the validation cohort

Variables	Univariate analyses				Multivariate analyses			
	Hazard Ratio	95% CI	P value	n*	Hazard Ratio	95% CI	P value	n*
**Clinic Findings**								
Age	0.72	0.55-0.94	1.50E-02	586	0.81	0.61-1.06	1.20E-01	562
LN	0.63	0.43-0.93	2.10E-02	1052	0.79	0.53-1.19	2.70E-01	562
Size	1.79	1.41-2.27	1.40E-06	772	1.49	1.13-1.96	4.20E-03	562
Grade	2.37	1.67-3.37	1.50E-06	754	1.68	1.12-2.52	1.30E-02	562
ER	1.47	1.18-1.83	5.20E-04	1058	0.94	0.59-1.50	8.00E-01	562
Her2	0.71	0.55-0.90	5.70E-03	1058	0.73	0.49-1.07	1.10E-01	562
TN	1.43	1.10-1.85	6.60E-03	1058	1.18	0.67-2.08	5.70E-01	562
Her2+/TN	1.56	1.27-1.91	2.20E-05	1058	1.35	0.91-2.00	1.30E-01	562
CMTC	2.40	1.88-3.05	1.20E-12	1058	1.73	1.23-2.44	1.90E-03	562
**Gene Signatures†**								
37GS	1.27	1.03-1.57	2.70E-02	1058	0.70	0.54-0.90	6.00E-03	1058
70GS	1.39	1.13-1.71	2.20E-03	1058	1.17	0.94-1.45	1.60E-01	1058
76GS	1.96	1.60-2.39	4.60E-11	1058	1.35	1.06-1.73	1.70E-02	1058
97GS	2.07	1.69-2.54	1.50E-12	1058	1.20	0.82-1.74	3.50E-01	1058
ERGS	1.89	1.54-2.32	9.70E-10	1058	1.14	0.79-1.64	4.90E-01	1058
ESGS	1.88	1.53-2.30	1.20E-09	1058	1.11	0.84-1.48	4.50E-01	1058
IGS	1.99	1.57-2.53	1.60E-08	1058	1.23	0.88-1.72	2.20E-01	1058
P53GS	1.69	1.34-2.12	6.10E-06	1058	1.13	0.83-1.53	4.40E-01	1058
PAM50	1.66	1.35-2.05	1.60E-06	1058	1.10	0.82-1.48	5.40E-01	1058
Proliferation	1.80	1.47-2.19	1.10E-08	1058	1.18	0.92-1.50	1.90E-01	1058
SDPP	1.80	1.47-2.20	1.20E-08	1058	1.11	0.83-1.48	5.00E-01	1058
Subtype	1.37	1.12-1.68	2.00E-03	1058	0.69	0.51-0.93	1.50E-02	1058
TGFβRII	1.00	0.81-1.23	1.00E+00	1058	0.74	0.59-0.92	7.10E-03	1058
WS	2.24	1.61-3.10	1.50E-06	1058	1.45	1.00-2.11	4.80E-02	1058
CMTC	2.40	1.88-3.05	1.20E-12	1058	1.43	1.00-2.04	4.90E-02	1058

*The number of cases on which the information of the specific variable is available in the validation cohort. †Tumors were dichotomized into good and poor prognosis groups based on 14 independent prognostic gene signatures and CMTC; for Subtype and PAM50, normal-like and luminal A were placed in good prognosis group, with luminal B, basal-like and Her2 status in poor prognosis group; CMTC-1 was in good prognosis group, with CMTC-2 and CMTC-3 in poor group. See Supplemental methods and Table S3 for detailed information on the gene signatures.
