# Supplementary material for: A new gene expression signature, the ClinicoMolecular Triad Classification, may improve prediction and prognostication of breast cancer at the time of diagnosis
Source: Breast Cancer Res. 2011 Sep 22;13(5):R92. doi: 10.1186/bcr3017 (PMC3262204; doi:10.1186/bcr3017)
Supplement: Additional file 8 — Supplementary Table S6 Association between relapse-free survivals and Her2+/TN status. Fourteen gene signatures and CMTC in the seven hundred fifty-six ER+ breast cancer patients with or without ET. ER = estrogen receptor; ERGS = estrogen-regulated gene expression signature; ESGS = embryonic stem cell-like gene signature; PAM50 = 50-gene prediction analysis of microarray; SDPP = stroma-derived prognostic predictor; TGFβRII = transforming growth factor β receptor type II; TN = triple-negative; WS = wound-response gene signature. [file bcr3017-S8.PDF]

Table S6 Association between relapse-free survivals and Her2+/TN status, 14 gene signatures and CMTC in the 756 ER+ breast cancer patients with or without endocrine therapy (ET)

Prognostic classifiers	Prognosis of classifiers in the 756 patients*			Prognosis of ET in the good prognosis patients		
	No. in good prognosis	Chi square	*P* value	No. in endocrine therapy	Chi square	*P* value
Her2+/TN	641	8.7800	3.00E-03	338	0.0002	9.89E-01
37GS	326	9.5950	2.00E-03	166	0.8891	3.46E-01
70GS	141	10.5100	1.20E-03	44	0.1554	6.93E-01
76GS	497	21.4900	3.54E-06	244	0.7537	3.85E-01
97GS	487	47.1900	6.42E-12	228	1.0530	3.05E-01
ERGS	433	40.3100	2.15E-10	198	1.4550	2.28E-01
ESGS	430	18.9400	1.35E-05	194	0.0041	9.49E-01
IGS	283	23.1000	1.53E-06	130	0.0449	8.32E-01
P53GS	313	26.5700	2.54E-07	146	0.4157	5.19E-01
PAM50	340	29.3400	6.05E-08	157	0.0142	9.05E-01
Proliferation	485	19.6900	9.09E-06	228	0.4680	4.94E-01
SDPP	518	18.0000	2.21E-05	242	0.0254	8.74E-01
Subtype	433	11.9200	6.00E-04	210	0.2257	6.35E-01
TGFβRII	444	0.0026	9.59E-01	212	0.0214	8.84E-01
WS	151	20.1000	7.35E-06	49	0.4940	4.82E-01
CMTC	299	37.5400	8.94E-10	115	5.0780	2.42E-02

*See Supplemental methods and Table S3 for details on how each tumor is classified into either a good or a poor prognosis group by individual gene signatures. The Chi square and *P* values were determined by Log-rank Test.
