# Supplementary material for: A new gene expression signature, the ClinicoMolecular Triad Classification, may improve prediction and prognostication of breast cancer at the time of diagnosis
Source: Breast Cancer Res. 2011 Sep 22;13(5):R92. doi: 10.1186/bcr3017 (PMC3262204; doi:10.1186/bcr3017)
Supplement: Additional file 9 — Supplementary Figure S2 Benefits of ET in CMTC-1 ER+ breast cancer at different cancer stages. Kaplan-Meier analyses were used to compare relapse-free survival between ET-treated and no-treatment ER+ breast cancer (A) in 155 stage I CMTC-1 cancers and (B) in 142 stage II or worse (stage II+) CMTC-1 cancers. The P values were determined by using the log-rank test. CMTC = ClinicoMolecular Triad Classification; ER = estrogen receptor; ET = endocrine therapy. [file bcr3017-S9.PDF]

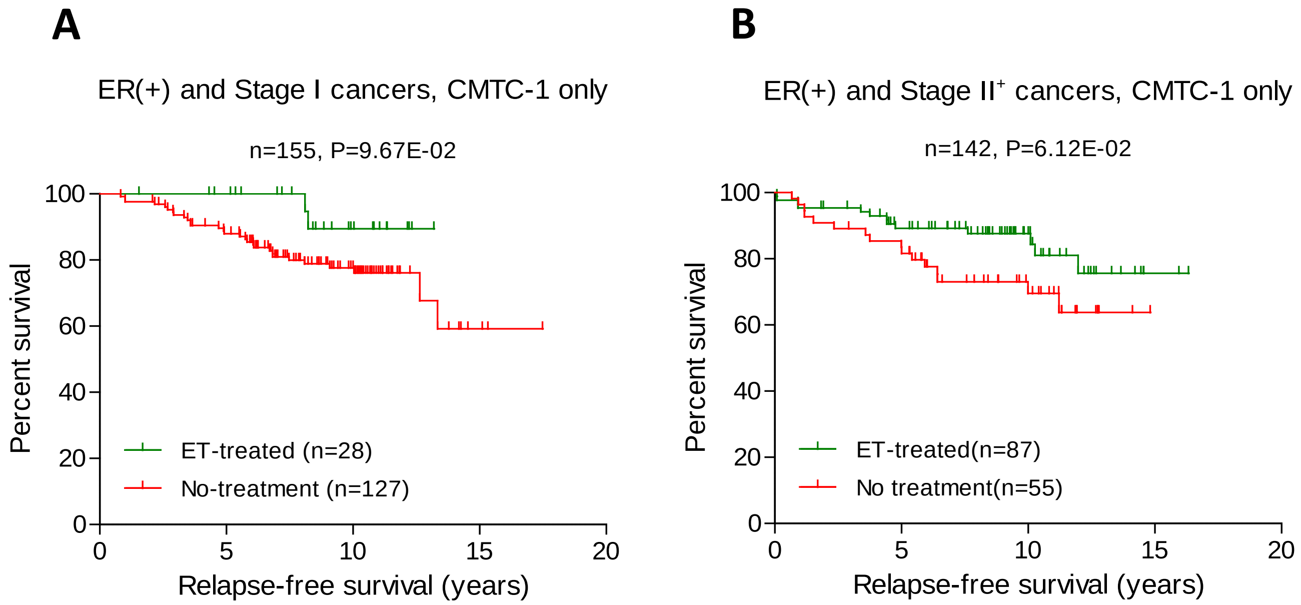

Figure S2 The benefits of endocrine therapy (ET) in CMTC-1 ER+ breast cancers at different cancer stages. Kaplan-Meier analyses were used to compare relapse-free survivals between ET-treated and no treatment ER+ breast cancers in 155 stage I CMTC-1 cancers (**A**), and in 142 stage 2 or worse (stage II+) CMTC-1 cancers (**B**). The *P* values were determined by Log-rank test.
