# Supplementary material for: A new gene expression signature, the ClinicoMolecular Triad Classification, may improve prediction and prognostication of breast cancer at the time of diagnosis
Source: Breast Cancer Res. 2011 Sep 22;13(5):R92. doi: 10.1186/bcr3017 (PMC3262204; doi:10.1186/bcr3017)
Supplement: Additional file 10 — Supplementary Table S7 Receiver operating characteristic analysis of the ability of independent gene expression signatures to predict pathological complete responses in breast cancer treated with neoadjuvant chemotherapy. CI = confidence interval; CMTC = ClinicoMolecular Triad Classification; ERGS = estrogen-regulated gene expression signature; ESGS = embryonic stem cell-like gene signature; Her2 = human epidermal growth factor receptor 2; IGS = "invasiveness" gene signature; LumA = luminal A; LumB = luminal B; PAM50 = 50-gene prediction analysis of microarray; SDPP = stroma-derived prognostic predictor; TGFβRII = transforming growth factor β receptor type II; TN = triple-negative; WS = wound-response gene signature. [file bcr3017-S10.PDF]

Table S7 Receiver operating characteristic analysis of the ability of independent gene expression signatures to predict pathological complete responses in breast cancers with neoadjuvant chemotherapy

Gene Signatures	All cancers (n=248)			Her2+/TN cancers (n=111)		
	AUC	95% CI	*P* value	AUC	95% CI	*P* value
37GS	0.615	0.53-0.70	1.19E-02	0.574	0.47-0.68	1.93E-01
70GS	0.634	0.56-0.71	3.54E-03	0.597	0.49-0.70	8.94E-02
76GS	0.578	0.49-0.66	8.81E-02	0.546	0.43-0.66	4.23E-01
97GS	0.747	0.67-0.82	6.79E-08	0.633	0.53-0.74	1.93E-02
ERGS	0.735	0.66-0.81	3.01E-07	0.619	0.51-0.73	3.72E-02
ESGS	0.693	0.62-0.77	2.45E-05	0.642	0.54-0.74	1.29E-02
IGS	0.713	0.64-0.79	3.18E-06	0.626	0.52-0.73	2.72E-02
P53GS	0.715	0.64-0.79	2.57E-06	0.551	0.44-0.66	3.72E-01
PAM50-Basal	0.801	0.74-0.86	4.83E-11	0.666	0.56-0.77	3.67E-03
PAM50-Her2	0.694	0.61-0.78	2.30E-05	0.583	0.47-0.70	1.46E-01
PAM50-LumA	0.798	0.74-0.86	7.58E-11	0.657	0.56-0.76	5.86E-03
PAM50-LumB	0.715	0.64-0.79	2.55E-06	0.598	0.49-0.71	8.49E-02
PAM50-Normal	0.600	0.51-0.69	2.97E-02	0.555	0.44-0.67	3.34E-01
Proliferation	0.675	0.60-0.75	1.29E-04	0.588	0.48-0.69	1.22E-01
SDPP	0.767	0.69-0.84	5.53E-09	0.622	0.51-0.73	3.30E-02
Subtype-Basal	0.775	0.71-0.84	1.82E-09	0.641	0.54-0.75	1.32E-02
Subtype-Her2	0.780	0.72-0.84	9.66E-10	0.640	0.54-0.74	1.40E-02
Subtype-LumA	0.795	0.73-0.86	1.11E-10	0.666	0.56-0.77	3.56E-03
Subtype-LumB	0.675	0.60-0.75	1.31E-04	0.630	0.52-0.74	2.27E-02
Subtype-Normal	0.530	0.44-0.62	5.09E-01	0.554	0.34-0.55	3.47E-01
TGFβIIR	0.548	0.46-0.64	2.90E-01	0.506	0.40-0.62	9.22E-01
WS	0.659	0.58-0.74	5.19E-04	0.580	0.47-0.69	1.62E-01
CMTC1	0.790	0.72-0.86	2.52E-10	0.675	0.57-0.78	2.21E-03
CMTC2	0.756	0.68-0.83	2.20E-08	0.632	0.52-0.74	2.06E-02
CMTC3	0.811	0.75-0.88	1.08E-11	0.718	0.62-0.81	1.29E-04

AUC, Area Under the Curve. See Supplemental methods and Table S3 for detailed information on the gene signatures.
