# Supplementary material for: A new gene expression signature, the ClinicoMolecular Triad Classification, may improve prediction and prognostication of breast cancer at the time of diagnosis
Source: Breast Cancer Res. 2011 Sep 22;13(5):R92. doi: 10.1186/bcr3017 (PMC3262204; doi:10.1186/bcr3017)
Supplement: Additional file 11 — Supplemental Table S8 The prediction of pCRs in 248 breast cancer patients treated with neoadjuvant chemotherapy on the basis of CMTC and 14 independent prognostic gene expression signatures. CMTC = ClinicoMolecular Triad Classification; PAM50 = 50-gene prediction analysis of microarray; SDPP = stroma-derived prognostic predictor; TGFβRII = transforming growth factor β receptor type II; WS = wound-response gene signature. [file bcr3017-S11.PDF]

**Table S8 The prediction of pathological complete responses (pCR) in 248 breast cancer patients with neoadjuvant chemotherapy by CMTC and 14 independent prognostic gene expression signatures**

| Signatures | Sensitivity | Specificity | PPV | NPV | Acc |
|---|---|---|---|---|---|
| 37GS | 58.0 | 63.6 | 28.7 | 85.7 | 62.5 |
| 70GS | 98.0 | 20.2 | 23.7 | 97.6 | 35.9 |
| 76GS | 46.0 | 68.2 | 26.7 | 83.3 | 63.7 |
| 97GS | 78.0 | 57.1 | 31.5 | 91.1 | 61.3 |
| ERGS | 88.0 | 45.5 | 28.9 | 93.8 | 54.0 |
| ESGS | 74.0 | 62.6 | 33.3 | 90.5 | 64.9 |
| IGS | 96.0 | 30.8 | 25.9 | 96.8 | 44.0 |
| P53GS | 94.0 | 28.8 | 25.0 | 95.0 | 41.9 |
| PAM50 | 82.0 | 68.7 | 39.8 | 93.8 | 71.4 |
| Proliferation | 56.0 | 67.2 | 30.1 | 85.8 | 64.9 |
| SDPP | 74.0 | 70.7 | 38.9 | 91.5 | 71.4 |
| Subtype | 76.0 | 73.2 | 41.8 | 92.4 | 73.8 |
| TGFβIIR | 48.0 | 58.6 | 22.6 | 81.7 | 56.5 |
| WS | 94.0 | 15.7 | 22.0 | 91.2 | 31.5 |
| CMTC | 78.0 | 72.7 | 41.9 | 92.9 | 73.8 |

The percentages in sensitivity, specificity, positive predictive value (PPV), negative predictive value (NPV) and diagnostic accuracy (Acc). Tumors were dichotomized into good and poor prognosis groups on all two-class prognostic gene signatures, and the poor groups were used to predict pCR. For Subtype and PAM50 signatures, basal-like and Her2 status were grouped to predict pCR and compared to normal-like, luminal A and luminal B subtypes; CMTC-3 was used to predict pCR and to compare with CMTC-1 and CMTC-2 groups. See Supplemental methods and Table S3 for detailed information on the gene signatures.
